# An EBV-related CD4 TCR immunotherapy inhibits tumor growth in an HLA-DP5^+^ nasopharyngeal cancer mouse model

**DOI:** 10.1172/JCI172092

**Published:** 2024-02-27

**Authors:** Chenwei Wang, Jiewen Chen, Jingyao Li, Zhihong Xu, Lihong Huang, Qian Zhao, Lei Chen, Xiaolong Liang, Hai Hu, Gang Li, Chengjie Xiong, Bin Wu, Hua You, Danyi Du, Xiaoling Wang, Hongle Li, Zibing Wang, Lin Chen

**Affiliations:** 1 Guangzhou Medical University–Guangzhou Institute of Biomedicine and Health (GMU-GIBH) Joint School of Life Sciences and Guangdong–Hong Kong–Macau Joint Laboratory for Cell Fate Regulation and Diseases, Guangzhou Medical University, Guangzhou, Guangdong, China.; 2 State Key Laboratory of Chemical Biology and Drug Discovery, Department of Applied Biology and Chemical Technology, The Hong Kong Polytechnic University, Hong Kong, China.; 3 Laboratory for Synthetic Chemistry and Chemical Biology Limited, Hong Kong, China.; 4 Department of Pathology, Air Force Hospital of Southern Theater Command, Guangzhou, Guangdong, China.; 5 Department of Otolaryngology–Head and Neck Surgery, Huiqiao Medicine Center, Nanfang Hospital, Southern Medical University, Guangzhou, Guangdong, China.; 6 Laboratory for Excellence in Systems Biomedicine of Pediatric Oncology, Department of Pediatric Hematology and Oncology, Children’s Hospital of Chongqing Medical University, Chongqing, China.; 7 Department of Otolaryngology–Head and Neck Surgery, Precision Medical Center, Nanfang Hospital, Southern Medical University, Guangzhou, Guangdong, China.; 8 Department of Immunotherapy, The Affiliated Cancer Hospital of Zhengzhou University & Henan Cancer Hospital, Zhengzhou, Henan, China.

**Keywords:** Immunology, Cancer immunotherapy, T cell receptor

## Abstract

Adoptive transfer of T cell receptor–engineered T cells (TCR-T) is a promising strategy for immunotherapy against solid tumors. However, the potential of CD4^^^^+^^^^ T cells in mediating tumor regression has been neglected. Nasopharyngeal cancer is consistently associated with EBV. Here, to evaluate the therapeutic potential of CD4 TCR-T in nasopharyngeal cancer, we screened for CD4 TCRs recognizing EBV nuclear antigen 1 (EBNA1) presented by HLA-DP5. Using mass spectrometry, we identified EBNA1____567–581____, a peptide naturally processed and presented by HLA-DP5. We isolated TCR135, a CD4 TCR with high functional avidity, that can function in both CD4^^^^+^^^^ and CD8^^^^+^^^^ T cells and recognizes HLA-DP5–restricted EBNA1____567–581____. TCR135-transduced T cells functioned in two ways: directly killing HLA-DP5^^^^+^^^^EBNA1^^^^+^^^^ tumor cells after recognizing EBNA1 presented by tumor cells and indirectly killing HLA-DP5–negative tumor cells after recognizing EBNA1 presented by antigen-presenting cells. TCR135-transduced T cells preferentially infiltrated into the tumor microenvironment and significantly inhibited tumor growth in xenograft nasopharyngeal tumor models. Additionally, we found that 62% of nasopharyngeal cancer patients showed 50%–100% expression of HLA-DP on tumor cells, indicating that nasopharyngeal cancer is well suited for CD4 TCR-T therapy. These findings suggest that TCR135 may provide a new strategy for EBV-related nasopharyngeal cancer immunotherapy in HLA-DP5^^^^+^^^^ patients.

## Abstract 2

A CD4+ T cell receptor specific to HLA-DP5-restricted EBNA1_567-581_, provides a strategy for Epstein-Barr virus-related nasopharyngeal cancer immunotherapy in HLA-DP5^+^ patients.

## Graphical abstract

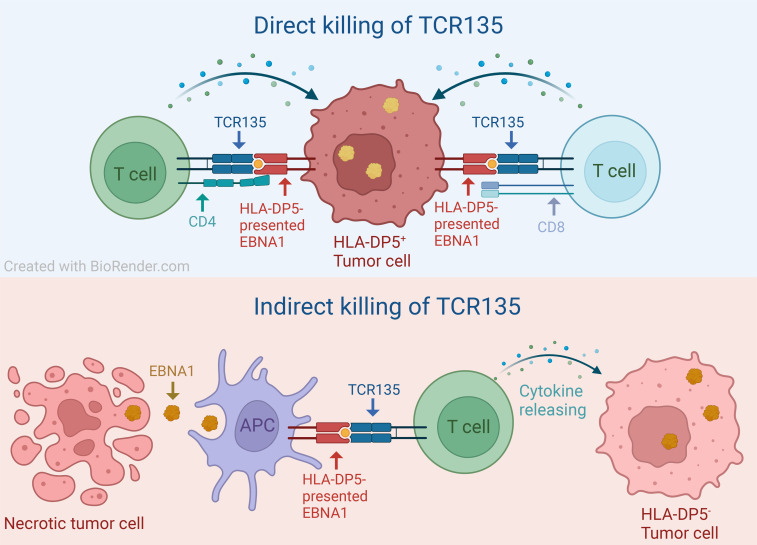

## Introduction

Recent advances in immunotherapy have shown promising results in the treatment of solid tumors. Adoptive transfer of T cell receptor–engineered (TCR-engineered) T cells (TCR-T) is a promising approach that has garnered much attention in this regard. While most studies have focused on CD8^+^ T cells, the importance of CD4^+^ T cells in mediating antitumor response is increasingly recognized. CD8^+^ cytotoxic T lymphocytes are considered the primary mediators of tumor cell killing, but CD4^+^ T cells are also critical for the generation of robust antitumor immunity (1–3). Vaccination with CD4^+^ immunogenic neoepitopes induces cytotoxic T lymphocyte responses and confers antitumor activity in both mice and humans, highlighting the participation of CD4^+^ T cells in immunotherapy (4, 5). CD4^+^ T cells are also required in immune checkpoint therapy (6). Moreover, several studies have shown that the adoptive transfer of CD4^^^^+^^^^ T cells can induce tumor regression in mouse models (7–9).

CD4 TCR-engineered T cells have shown great potential for the treatment of solid tumors. CD4 TCR-engineered T cells targeting MAGE-A3 (10), NY-ESO-1 (11), hTERT (12), and mutant KRAS (13) have been developed and proved to be able to kill antigen-expressing tumors in preclinical models. The efficacy of CD4^+^ T cells in treating tumors has also been demonstrated in clinical trials. In 2008, a patient with refractory metastatic melanoma achieved long-term complete remission after receiving autologous NY-ESO-1–specific CD4^+^ T cells (14). This promising result was followed by the report in 2014 of a patient with metastatic cholangiocarcinoma who achieved partial remission after treatment with CD4^+^ T cells targeting mutant ERBB2 (15). Furthermore, a phase I clinical trial of CD4 TCR-engineered T cells targeting MAGE-A3 observed objective remission in 3 of 9 patients in the high-dose group (16).

EBV is implicated in the pathogenesis of multiple malignancies, including B cell lymphoma, nasopharyngeal carcinoma (NPC), and gastric cancer (17). EBV nuclear antigen 1 (EBNA1) is one of the first viral proteins expressed after infection and is the only latent protein consistently expressed in all EBV-associated tumors (18). EBNA1-specific CD4^+^ T cells recognize and kill EBV-transformed lymphoblastoid cell lines and Burkitt’s lymphoma cells (19–22). Moreover, there are abundant CD4 epitopes restricted by a range of different HLA class II alleles located at the C-terminal half of EBNA1 (23). Tsang et al. (24) analyzed CD4^+^ T cell responses to EBNA1 in 78 healthy Chinese donors and found that HLA-DP5–restricted EBNA1_564–583_ was recognized in 47% (37/78) of donors tested.

Undifferentiated NPC is the cancer most consistently associated with EBV; latent EBV occurs in more than 95% of cases (25). Thus, we aimed to isolate CD4 TCR specific for HLA-DP5–restricted EBNA1_564–583_ and to evaluate its therapeutic potential in NPC. In this study, we identified TCR135, a CD4 TCR with high functional avidity that recognizes HLA-DP5–restricted EBNA1 in both CD4^+^ and CD8^+^ T cells in an HLA-DP–dependent manner. TCR135-transduced T cells significantly inhibited tumor growth in vivo in the xenograft NPC tumor model. Our findings suggest that TCR135 may provide a new strategy for immunotherapy treatment in HLA-DP5^+^ NPC patients.

## Results

### Isolation of HLA-DP5–restricted EBNA1 TCR from healthy donors.

We used the pMHC II–hIgG1 heterodimerization strategy developed by Serra et al. (26) to produce the empty HLA-DP5 monomer (Supplemental Figure 1A; supplemental material available online with this article; https://doi.org/10.1172/JCI172092DS1). The soluble HLA-DP5 monomer was successfully purified (Supplemental Figure 1B) and recognized by the anti–HLA-DP B7/21 antibody, indicating that the HLA-DP5 monomer retained the native conformation of HLA-DP5 (Supplemental Figure 1C). The EBNA1_564–583_ peptide–loaded HLA-DP5 monomers were then used to generate tetramers.

**Figure 1:**
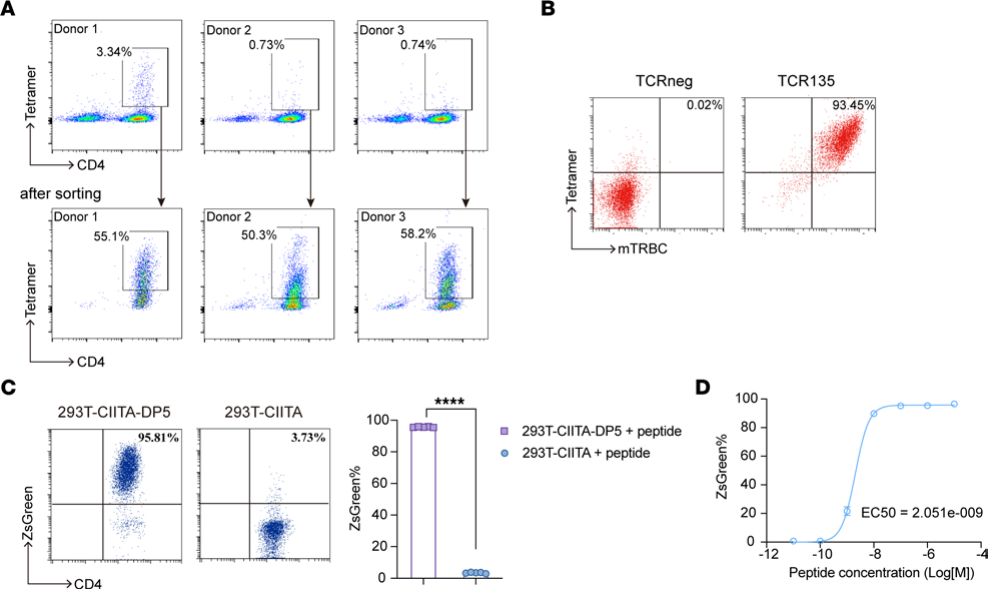
Isolation of HLA-DP5–restricted EBNA1 TCR from healthy donors. (**A**) Naive CD4^+^ T cells were isolated from HLA-DP5^+^ healthy donors and stimulated by autologous DCs pulsed with the EBNA1_564–583_ peptide. The representative tetramer staining dot plots and the percentage of tetramer-positive cells in stimulated T cells are shown. (**B**) TCR expression on the cell surface of TCR135-transduced JK4NF cells was assessed by labeling with anti–mouse TCRβ (mTRBC) and EBNA1_564–583_ tetramer (Tetramer). Non-transduced JK4NF cells (TCRneg) were included as negative control. (**C**) Flow cytometry assays of ZsGreen expression in TCR135-transduced JK4NF cells cocultured overnight with EBNA1_564–583_ peptide–loaded 293T-CIITA-DP5 or 293T-CIITA cells. 2-tailed unpaired Student’s *t* test, ****P < 0.0001.(**D**) The functional avidity assay of the TCR135. TCR135-transduced JK4NF cells were cocultured with 293T-CIITA-DP5 cells pulsed with the EBNA1_564–583_ peptide at the indicated concentration overnight. The frequency of ZsGreen-expressing cells was measured by flow cytometry. EC_50_ value was determined using a nonlinear regression curve.

We used the EBNA1_564–583_ peptide to stimulate CD4^+^ T cells in PBMCs isolated from HLA-DP5^+^ healthy donors. After approximately 14–21 days in culture, stimulated T cells were stained with the DP5/EBNA1_564–583_ tetramer. CD4^+^tetramer^+^ T cells were successfully enriched in 5 of 6 healthy donors. The representative CD4^+^tetramer^+^ T cells in donors 1–3 are shown in Figure 1A. After stimulation, single CD4^+^tetramer^+^ T cells were sorted into 96-well plates and amplified using single-cell PCR to obtain TCRs. In total, 22 EBNA1-specific TCRs were identified. Among them, TCR135 displayed the highest avidity and was selected for further investigation in subsequent studies.

To evaluate the functional avidity of CD4 TCRs, we transduced J.RT3-T3.5, a derivative of the E6-1 clone of Jurkat lacking the TCR β chain, with genes encoding CD4 and the NFAT-ZsGreen reporter (referred to as JK4NF cells below). TCR135-transduced JK4NF cells displayed expression of the mouse TCR β chain constant region (mTRBC) and binding to the DP5/EBNA1_564–583_ tetramer (Figure 1B). JK4NF cells transduced with TCR135 strongly recognized HLA-DP5^+^ 293T-CIITA-DP5 cells pulsed with the EBNA1_564–583_ peptide, but not 293T-CIITA cells pulsed with the peptide (Figure 1C). The titration of the EBNA1_564–583_ peptide indicated that TCR135-transduced JK4NF cells could be activated by 293T-CIITA-DP5 cells pulsed with a minimum of 1 nM of the target peptide, with a mean EC_50_ of approximately 2.05 nM (Figure 1D).

### EBNA1_564–583_ was endogenously processed by HLA-DPA1*02:02/DPB1*0501 but not by HLA-DPA1*01:03/DPB1*0501.

**Figure 2:**
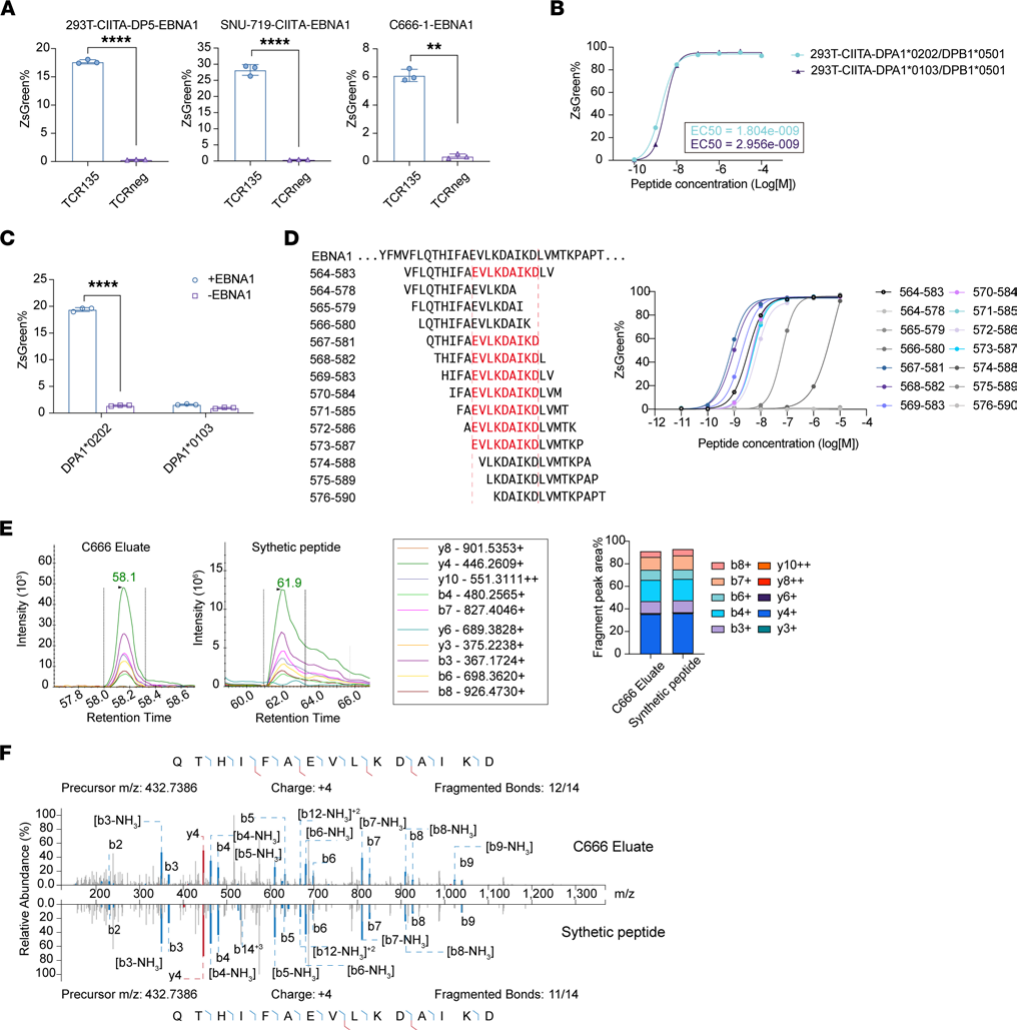
Identification of the endogenously presented epitope of EBNA1_564–583_. (**A**) The percentage of ZsGreen expression in TCR135-transduced (TCR135) or non-transduced (TCRneg) JK4NF cells after coculture with 293T-CIITA-DP5-EBNA1, SNU-719-CIITA-EBNA1, or C666-1-EBNA1 tumor cells. 2-tailed unpaired Student’s *t* test, **P < 0.01, ****P < 0.0001. (**B**) The percentage of ZsGreen expression in TCR135-transduced JK4NF cells after coculture with the EBNA1_564–583_ peptide–pulsed DPA1*0202/DPB1*0501– or DPA1*0103/DPB1*0501–positive cells. EC_50_ values were determined using a nonlinear regression curve. (**C**) The percentage of ZsGreen expression in TCR135-transduced JK4NF cells after coculture with 293T-CIITA-DPA1*0202/DPB1*0501 or 293T-CIITA-DPA1*0103/DPB1*0501 transduced with (+EBNA1) or without (–EBNA1) EBNA1. 2-way ANOVA and Šidák’s multiple comparisons, ****P < 0.0001. (**D**) Identification of the minimal epitope of the EBNA1_564–583_ peptide recognized by TCR135. The right panel shows the functional avidity curve of TCR135-transduced JK4NF cells cocultured with 293T-CIITA-DP5 cells pulsed with the indicated overlapping peptides. The left panel shows the amino acid sequences of overlapping peptides. The red text shows the minimal epitope EBNA1_573–581_ recognized by TCR135. (**E**) The distribution of fragment ion intensity derived from EBNA1_567–581_ eluted from C666-1-EBNA1_C–terminus_ cells (C666 Eluate) and its synthetic peptide. (**F**) Mirror plot displaying the MS2 spectra of the EBNA1_567–581_ eluted from C666-1-EBNA1_C–terminus_ cells (C666 Eluate) and its synthetic peptide. Peaks represent *b* ions in blue and *y* ions in red.

We next investigated whether EBNA1_564–583_ could be endogenously processed by tumor cells. Full-length EBNA1 was overexpressed in 293T-CIITA-DP5 cells (293T-CIITA-DP5-EBNA1) and cocultured with TCR135-transduced JK4NF cells or TCR-negative (TCRneg) JK4NF cells. As shown in Figure 2A, TCR135-transduced JK4NF cells but not TCRneg cells were activated by 293T-CIITA-DP5-EBNA1 cells. The EBV-associated gastric tubular adenocarcinoma cell line SNU-719 and the nasopharyngeal carcinoma cell line C666-1 were also investigated. Both cell lines have the HLA-DP genotype HLA-DPA1*02:02/DPB1*0501 (Supplemental Figure 2A). C666-1 cells naturally expressed HLA-DP on the cell surface as indicated by anti–HLA-DP antibody B7/21 flow cytometry analysis (Supplemental Figure 2B). CIITA overexpression also successfully induced HLA-DP expression on the SNU-719-CIITA cell surface (Supplemental Figure 2B). Most nasopharyngeal carcinoma cell lines have lost the EBV genome during the in vitro establishment of cell lines from biopsies or xenografts (27, 28). It has been reported that even when the EBV genome remains intact during establishment, with culturing of 293-EBV cells in vitro, the EBV genome is gradually lost from cells unless under selection because clones without the EBV genome have greater proliferation ability (29). Although both SNU-719 and C666-1 cell lines have been reported to contain a complete EBV genome (30, 31), we found that the expression level of *EBNA1* mRNA in SNU-719 and C666-1 cell lines was quite low, less than 10% of that in the lymphoblastoid cell lines (Supplemental Figure 2C). Therefore, full-length *EBNA1* was also transduced into SNU-719-CIITA and C666-1 cell lines. As shown in Figure 2A, TCR135-transduced JK4NF cells were significantly activated by SNU-719-CIITA-EBNA1 and C666-1-EBNA1 cells. TCR135 could also be activated by tumor cells overexpressing C-terminus EBNA1 (Supplemental Figure 2D). These results indicate that EBNA1_564–583_ can be endogenously processed and presented by HLA-DP5^+^EBNA1^+^ cells.

The *HLA-DPA1* gene displayed significantly less polymorphism than the *HLA-DPB1* gene, with DPA1*0202 and DPA1*0103 being the 2 most common genotypes (32). We wondered whether EBNA1_564–583_ could also be endogenously processed by HLA-DPA1*01:03/DPB1*0501. Therefore, 293T-CIITA cells overexpressing DPA1*01:03/DPB1*0501 were constructed. Exogenous EBNA1_564–583_ peptide–loaded 293T-CIITA-DPA1*01:03/DPB1*0501 cells showed slightly weaker but similar ability to activate TCR135 as compared with 293T-CIITA-DPA1*02:02/DPB1*0501 cells (Figure 2B). However, TCR135 did not recognize 293T-CIITA-DPA1*01:03/DPB1*0501 with endogenously expressed full-length EBNA1 (Figure 2C) or C-terminus EBNA1 (Supplemental Figure 2E). As a result, we concluded that although EBNA1_564–583_ peptide could be exogenously loaded onto HLA-DPA1*01:03/DPB1*0501, it cannot be endogenously presented by HLA-DPA1*01:03/DPB1*0501.

### EBNA1_567–581_ was presented on the cell surface and identified by mass spectrometry.

To identify the minimal epitope of the EBNA1_564–583_ peptide recognized by TCR135, overlapping 15-mer peptides shifted by 1 amino acid were individually loaded onto 293T-CIITA-DP5 cells. We found that EBNA1_573–581_ was the minimal epitope that could activate TCR135 (Figure 2D and Supplemental Figure 3). We next determined the sequences of endogenously processed EBNA1 peptides presented by HLA-DP5 using liquid chromatography with tandem mass spectrometry (LC-MS/MS). The peptide–HLA-DP molecules of C666-1 cells that express C-terminus EBNA1 were immunoprecipitated by the anti–HLA-DP B7/21 antibody. The eluted peptides bound to HLA-DP5 were analyzed by LC-MS/MS. The 20-mer EBNA1_564–583_ and 14- to 18-mer peptides containing EBNA1_573–581_ (see peptide sequences in Supplemental Table 1) were synthesized and used as standards during LC-MS/MS. Only EBNA1_567–581_ was identified in the eluted HLA-DP5 immunopeptidome (Figure 2E). The MS/MS spectrum of the eluted EBNA1_567–581_ peptide matched with that of the synthetic peptide (Figure 2F). We failed to detect the 20-mer EBNA1_564–583_ or any other 14- to 18-mer peptides containing EBNA1_573–581_. Interestingly, the EBNA1_567–581_ peptide also showed the highest functional avidity in peptide-binding assay (Figure 2D). These results indicate that EBNA1_567–581_ was the natural epitope of TCR135 that presented on the HLA-DP5^+^EBNA1^+^ cell surface.

### TCR135-engineered primary CD4^+^ T cells specifically recognized HLA-DP5^+^EBNA1^+^ tumor cells.

**Figure 3:**
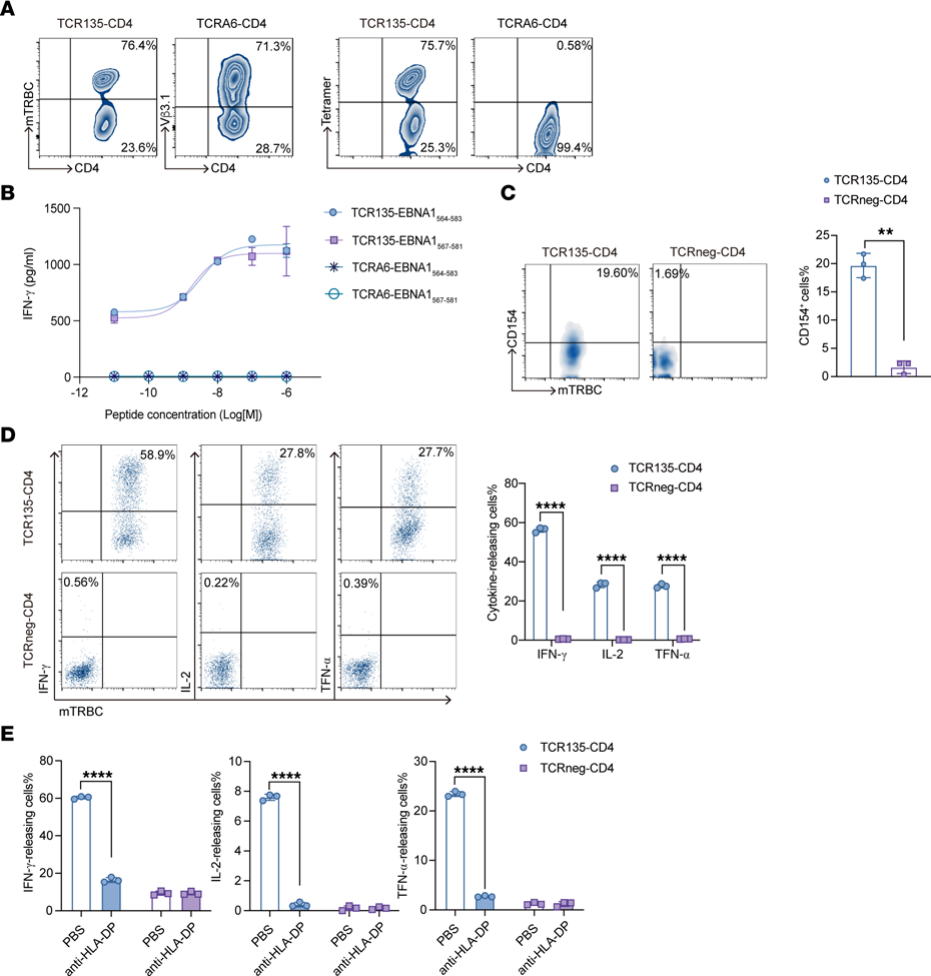
TCR135-engineered primary CD4^+^ T cells specifically recognized HLA-DP5^+^EBNA1^+^ tumor cells. (**A**) Flow cytometry analysis of the expression of TCR135 on TCR135-transduced (TCR135-CD4) or TCRA6-transduced (TCRA6-CD4) primary CD4^+^ T cells. T cells were stained with anti–mouse TCRβ (mTRBC) or EBNA1_564–583_ tetramer (Tetramer). The number is the percentage of positive cells in gated CD4^+^ T cells. (**B**) TCR135-CD4 T cells and TCRA6-CD4 T cells were cocultured with 293T-CIITA-DP5 cells pulsed with the EBNA1_564–583_ or EBNA1_567–581_ peptide at the indicated concentration overnight. IFN-γ secreted in the supernatant was detected by ELISA. (**C**) The TCR135-CD4 or TCRneg-CD4 T cells were cocultured overnight with C666-1-EBNA1 tumor cells, and then the percentage of CD154-releasing cells in CD4^+^ T cells was measured by flow cytometry. The right panel shows the statistical bar chart. 2-tailed unpaired Student’s *t* test, **P < 0.01. (**D**) Representative intracellular flow cytometry analysis (left panels) and a summary bar graph (right panel) for IFN-γ, IL-2, and TNF-α production in TCR135-CD4 or TCRneg-CD4 T cells after coculture with C666-1-EBNA1 cells. 2-tailed unpaired Student’s *t* test, ****P < 0.0001. (**E**) The frequency of IFN-γ–, IL-2–, and TNF-α–releasing cells of TCR135-CD4 or TCRneg-CD4 T cells cocultured with C666-1-EBNA1 cells with or without anti–HLA-DP antibody. 2-way ANOVA and Šidák’s multiple comparisons, ****P < 0.0001.

We further investigated the functionality of TCR135 in genetically engineered primary T cells. Naive CD4^+^ T cells from healthy donors were transduced with TCR135 lentiviral vectors (TCR135-CD4) or the irrelevant TCR (TCRA6) (TCRA6-CD4), which recognizes the HTLV-1 Tax peptide, as negative control, and then stained for the mouse β chain constant region and the DP5/EBNA1_564–583_ tetramer. As shown in Figure 3A, TCR135 was successfully expressed on the cell membrane of primary CD4^+^ T cells (for gating strategy see Supplemental Figure 4A).

TCR135-engineered CD4^+^ T cells (TCR135-CD4 cells) were cocultured with EBNA1_567–581_ or EBNA1_564–583_ peptide–pulsed 293T-CIITA-DP5 overnight, and released IFN-γ was detected by ELISA. TCR135-CD4 cells recognized exogenously pulsed peptides, but CD4^+^ T cells transduced with irrelevant TCR (TCRA6-CD4) did not (Figure 3B). TCR-T cells were then cocultured with C666-1-EBNA1 tumor cells to assess whether TCR135-CD4 cells could recognize the endogenously processed antigen. CD154 staining showed that TCR135-CD4 but not TCRneg-CD4 cells were activated by C666-1-EBNA1 tumor cells (Figure 3C). Similarly, intracellular cytokine staining showed that TCR135-CD4 cells recognized the naturally processed EBNA1 and expressed high levels of IFN-γ, IL-2, and TNF-α (Figure 3D; for gating strategy see Supplemental Figure 4B). Moreover, recognition of HLA-DP5^+^EBNA1^+^ tumor cells by TCR135-CD4 cells was successfully inhibited by antibody blockade of HLA-DP molecules, indicating that target recognition of TCR135 was HLA-DP restricted (Figure 3E).

### TCR135 also functions in primary CD8^+^ T cells.

**Figure 4:**
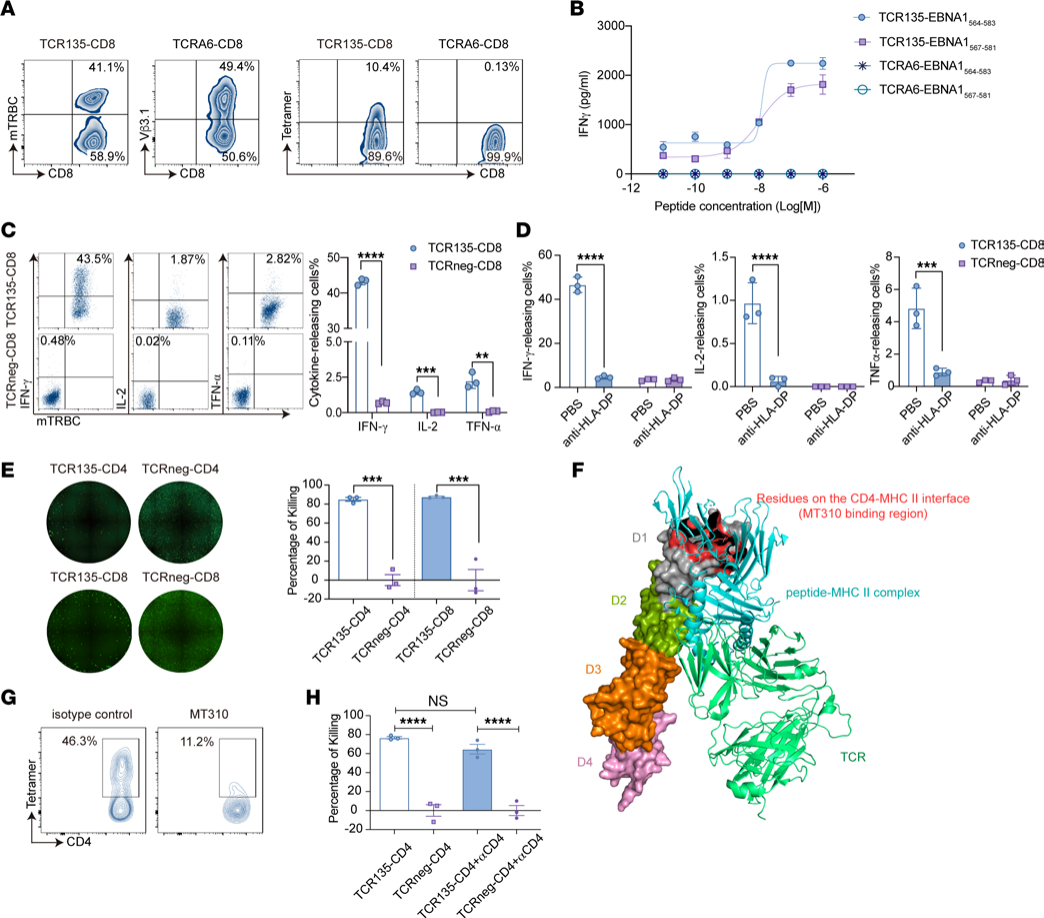
TCR135 could function in a CD4 coreceptor–independent manner. (**A**) Flow cytometry analysis of the expression of TCR135 on TCR135-transduced (TCR135-CD8) or TCRA6-transduced (TCRA6-CD8) primary CD8^+^ T cells. (**B**) TCR135-CD8 and TCRA6-CD8 cells were cocultured with 293T-CIITA-DP5 cells pulsed with the EBNA1_564–583_ or EBNA1_567–581_ peptide at the indicated concentration overnight. IFN-γ secreted in the supernatant was detected by ELISA. (**C**) Representative intracellular flow cytometry analysis (left panels) and a summary bar graph (right panel) for IFN-γ, IL-2, and TNF-α production in TCR135-CD8 or TCRneg-CD8 cells after coculture with C666-1-EBNA1 cells. 2-tailed unpaired Student’s *t* test, **P < 0.01, ***P < 0.001, ****P < 0.0001. (**D**) The frequency of IFN-γ–, IL-2–, and TNF-α–releasing cells of TCR135-CD8 or TCRneg-CD8 cells cocultured with C666-1-EBNA1 cells with or without anti–HLA-DP antibody. 2-way ANOVA and Šidák’s multiple comparisons, ***P < 0.001, ****P < 0.0001. (**E**) TCR135-CD4 or TCR135-CD8 cells were cocultured with C666-1-EBNA1 cells at an E/T ratio of 1:2 for 72 hours, and then the living tumor cells were analyzed by Celigo Image Cytometer fluorescence photography. The representative fluorescent images and statistical results are shown. 1-way ANOVA and Šidák’s multiple comparisons, ***P < 0.001. (**F**) The structure of a ternary complex of CD4, peptide–MHC II, and TCR (Protein Data Bank ID: 3T0E). The red surface indicates CD4 residues on the CD4–MHC II interface. D1–D4 indicates the 4 domains of CD4. (**G**) Tetramer staining of TCR135-transduced CD4^+^ T cells with or without CD4 antibody MT310. The number is the percentage of positive cells in gated CD4^+^ T cells. (**H**) TCR135-CD4 or TCRneg-CD4 cells were cocultured with C666-1-EBNA1 cells, with or without CD4 antibody clone MT310, for 72 hours. The percentage of cell killing and the statistical results are shown. 1-way ANOVA and Šidák’s multiple comparisons, ****P < 0.0001.

To analyze whether TCR135 could function in CD8^+^ T cells, naive CD8^+^ T cells from healthy donors were also transduced with TCR135 (TCR135-CD8) or irrelevant TCR (TCRA6) (TCRA6-CD8) lentiviral vectors. TCR135 was successfully expressed on the cell membrane of primary CD8^+^ T cells (Figure 4A). However, we noticed that the tetramer staining intensity was reduced on the surface of TCR135-CD8 T cells (Figure 4A). We then wondered whether TCR135 could function in CD8^+^ T cells. Thus, TCR-engineered CD8^+^ T cells were cocultured with EBNA1_567–581_ or EBNA1_564–583_ peptide–pulsed 293T-CIITA-DP5 overnight, and released IFN-γ was detected by ELISA. Surprisingly, TCR-engineered CD8^+^ T cells successfully recognized exogenously pulsed peptides but CD8^+^ T cells transduced with irrelevant TCR did not (Figure 4B). TCR135-engineered CD8^+^ T cells were also activated by C666-1-EBNA1 tumor cells and expressed higher levels of IFN-γ, IL-2, and TNF-α than non-transduced TCRneg-CD8 T cells (Figure 4C). An antibody blockade of HLA-DP molecules also prevented TCR135-CD8 cells from recognizing C666-1-EBNA1 tumor cells (Figure 4D). We next assessed the ability of TCR135-CD4 and TCR135-CD8 cells to kill tumor cells using Celigo Image Cytometer fluorescence photography. The growth of C666-1-EBNA1 tumor cells was significantly inhibited by both TCR135-CD4 and TCR135-CD8 cells (Figure 4E). These results demonstrated that TCR135 could also function in CD8^+^ T cells.

One function of the CD4 coreceptor is to stabilize the interaction of the TCR with MHC class II through binding to MHC class II with its membrane-distal D1 domain (33–35). The ability of TCR135 to function in CD8^+^ T cells implies that its interaction with HLA-DP5 was extremely robust, to the extent that it does not require the assistance of the D1 domain of a CD4 coreceptor.

To confirm these data further, we used the CD4-blocking antibody clone MT310. The MT310 antibody binds the complementarity-determining region 2–like (CDR2-like) region of CD4’s D1 domain (Figure 4F). Previous studies have shown that the MT310 antibody could block the binding of MHC class II tetramers to the cognate TCRs (36). Indeed, we observed a decrease in tetramer staining intensity on TCR135-CD4 cells when the MT310 antibody was present (Figure 4G). However, despite the presence of MT310 antibody, TCR135-CD4 cells still exhibited significant killing activity against C666-1-EBNA1 tumor cells (Figure 4H). Together, these results demonstrate that TCR135 could function in CD8^+^ T cells. This is due to the high affinity of TCR135, which eliminates the requirement for the CD4 coreceptor’s CDR2-like D1 domain.

We next assessed the ability of TCR135-engineered mixed CD4^+^ and CD8^+^ T cells (TCR135-T cells) to kill tumor cells. The growth of C666-1-EBNA1 tumor cells was significantly inhibited by TCR135-T cells (Supplemental Figure 5A). Moreover, TCR135-T cells inhibited the growth of C666-1-EBNA1 tumor cells at an effector-to-target ratio of 1:2 and 1:5, as assessed by real-time quantitative live-cell analysis platforms (Supplemental Figure 5B).

### TCR135-T cells mediated antitumor response in vivo.

**Figure 5:**
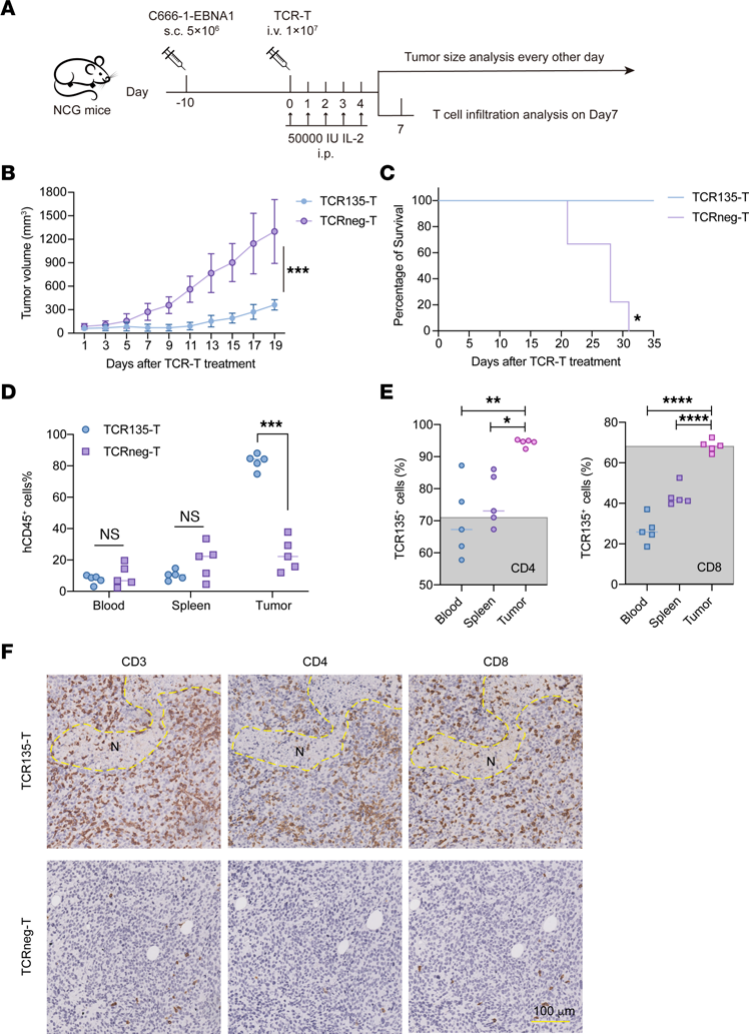
TCR135-T cells mediated antitumor response in vivo. (**A**) A schematic diagram of the animal experiments. C666-1-EBNA1 tumor cells were subcutaneously injected into NCG mice and then treated with TCR135-transduced T cells (*n* = 5) or TCRneg (*n* = 5) as control. (**B** and **C**) The tumor growth curve (**B**) and Kaplan-Meier survival curve (**C**) of mice are shown. Data are representative of 2 independent experiments. 2-way ANOVA test (**B**), log-rank (Mantel-Cox) test (**C**) *P < 0.05, ***P < 0.001. (**D**) The peripheral blood, spleen, and tumor samples were collected 7 days after the adoptive transfer of T cells. The percentage of hCD45 was analyzed by flow cytometry. 2-tailed unpaired Student’s *t* test, ***P < 0.001. (**E**) The percentage of TCR135^+^ cells in gated CD4^+^ (left) and CD8^+^ (right) T cells in peripheral blood, spleen, and tumor samples collected 7 days after the adoptive transfer of T cells. The gray shadow represents the percentage of TCR135^+^ cells in CD4^+^ or CD8^+^ T cells on day 0 before adoptive transfer. 1-way ANOVA and Dunnett’s multiple comparisons, *P < 0.05, **P < 0.01, ****P < 0.0001. (**F**) Tumor sections on day 7 after T cell injection were stained for CD3, CD4, and CD8 by immunohistochemistry. N, necrotic area. Scale bar: 100 μm.

To evaluate the therapeutic potential of TCR135-T cells in vivo, C666-1-EBNA1 tumor cells were subcutaneously injected into immunodeficient NCG mice (Figure 5A). The adoptive transfer of TCR135-T cells significantly inhibited tumor growth and greatly improved survival in all mice (Figure 5, B and C, and Supplemental Figure 6A). Additionally, the transfer of TCR135-T cells resulted in tumor regression in the SNU-719-CIITA-EBNA1 xenograft tumor model (Supplemental Figure 6, B and C).

We then collected peripheral blood, spleen, and tumor samples 7 days after T cell injection to examine TCR135-T cell infiltration in the C666-1-EBNA1 xenograft tumor model. While the percentage of cells positive for human CD45 (hCD45) in peripheral blood and spleen was similar between TCR135-transduced and TCRneg groups, there were significantly more hCD45^+^ cells in the tumor tissue of the TCR135-T group (Figure 5D). Interestingly, when we analyzed the percentage of TCR135^+^ cells in both CD4^+^ and CD8^+^ T cells, we found that the percentage of TCR135^+^ cells in peripheral blood and spleen was lower than the transduction rate of TCR135-T cells before adoptive transfer. However, the percentage of TCR135^+^ cells in tumor tissue was significantly higher than that in peripheral blood and spleen, and it was similar to the transduction rate of TCR135-T cells before adoptive transfer (Figure 5E). These data suggest TCR135^+^ cells preferentially distributed in tumor tissue.

Moreover, tumor tissue sections were analyzed by immunohistochemistry. Many CD3^+^, CD4^+^, or CD8^+^ T cells were detected in the tumor microenvironment of TCR135-T cell–treated mice, while only a few scattered positive signals were seen in the TCRneg-T group (Figure 5F). In addition, clear necrotic areas were present in the tumor tissue of the TCR135-T group but not in the TCRneg-T group. Together, these data demonstrate that TCR135-T cells successfully infiltrated the tumor microenvironment and mediated an antitumor response in vivo.

### Characteristics of TCR135-transduced T cells analyzed by scRNA-Seq.

**Figure 6:**
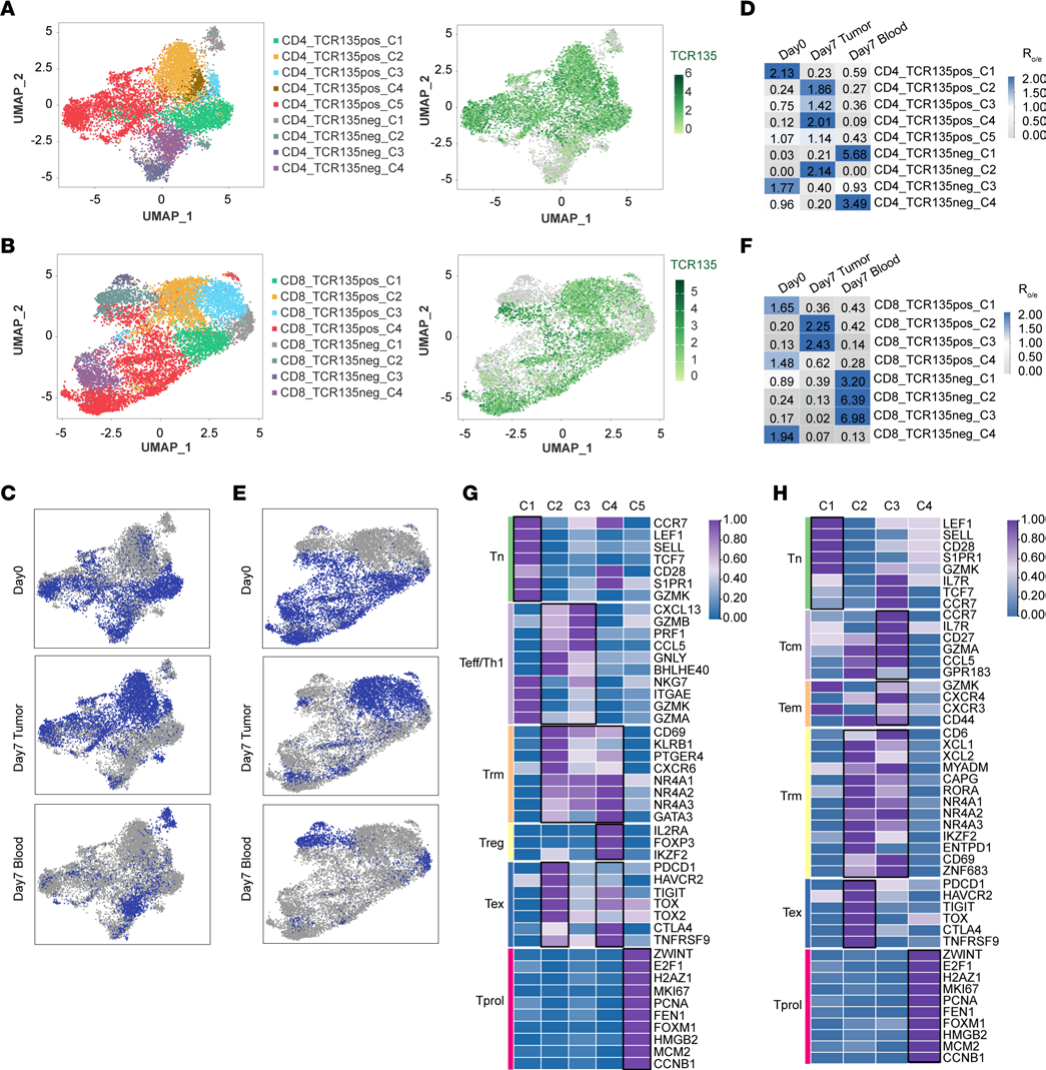
Characteristics of TCR135-transduced T cells analyzed by scRNA-Seq. scRNA-Seq was conducted on TCR135-T cells on day 0 before transplantation (Day0) and T cells sorted from tumor tissue (Day7 Tumor) and peripheral blood (Day7 Blood) on day 7 after transplantation. (**A** and **B**) Different clusters and expressions of the TCR135 gene in CD4^+^ (**A**) (*n* = 10,009) and CD8^+^ (**B**) (*n* = 9,727) T cells are shown by uniform manifold approximation and projection (UMAP). (**C**) CD4^+^ T cells from Day0 (*n* = 3,767), Day7 Tumor (*n* = 4,672), and Day7 Blood (*n* = 1,570) are highlighted in the UMAP plots. (**D**) Tissue preference of each cluster of CD4^+^ T cells estimated by R_o/e_ score. (**E**) CD8^+^ T cells from Day0 (*n* = 4,788), Day7 Tumor (*n* = 3,673), and Day7 Blood (*n* = 1,266) are highlighted in the UMAP plots. (**F**) Tissue preference of each cluster of CD8^+^ T cells estimated by R_o/e_ score. (**G** and **H**) Functional subsets of CD4^+^ TCR135pos (**G**) and CD8^+^ TCR135pos (**H**) T cell clusters defined by a set of known marker genes.

We performed single-cell RNA sequencing (scRNA-Seq) of TCR135-T cells on day 0 before transplantation into mice and of T cells isolated from mouse blood and tumor tissue on day 7 after transplantation. Based on the unsupervised clustering and the transcriptional level of the *TCR135* gene, we identified five TCR135-positive (TCR135pos) clusters and four TCR135-negative (TCR135neg) clusters in CD4^+^ T cells (Figure 6A), and four TCR135pos clusters and four TCR135neg clusters in CD8^+^ T cells (Figure 6B). Similar to the flow cytometry results, we found that TCR135pos clusters were enriched in tumor tissue and TCR135neg clusters were enriched in blood on day 7 after transplantation (Figure 6, C and E). The tissue distribution of each cluster was further confirmed by comparison of the observed and expected cell numbers of each cluster (R_o/e_) (37) (Figure 6, D and F). Partition-based graph abstraction (PAGA) trajectory analysis of CD4^+^ and CD8^+^ TCR135pos clusters confirmed a developmental trajectory from day 0 enriched clusters to day 7 tumor-enriched clusters (Supplemental Figure 7).

We then analyzed the transcriptional signatures of each TCR135pos cluster of CD4^+^ and CD8^+^ T cells. The cluster CD4_TCR135pos_C1 was enriched on day 0 before transplantation as indicated by the R_o/e_ index (Figure 6D) and showed a gene expression signature of naive T cells (Tn) (Figure 6G). Clusters CD4_TCR135pos_C2/C3/C4 were enriched in the tumor tissue at day 7 after transplantation (Day 7 Tumor) and showed the gene expression signatures of Th1 effector cells (Teff/Th1) (C2 and C3) and tissue-resident memory T cells (Trm) (C2, C3, and C4). The cluster CD4_TCR135pos_C4 were regulatory T cells (Treg) and expressed high levels of *Foxp3*. Clusters CD4_TCR135pos_C2/C4 showed gene expression signatures of exhausted T cells (Tex). Cluster CD4_TCR135pos_C5 was enriched in both day 0 and day 7 tumor samples and showed the signature of proliferating T cells (Tprol).

As with CD4^+^ cells, the cluster CD8_TCR135pos_C1 was enriched on day 0 before transplantation into mice as indicated by the R_o/e_ index (Figure 6E) and showed a gene expression signature of naive T cells (Tn) (Figure 6H). Clusters CD8_TCR135pos_C2/C3 were enriched in the day 7 tumor sample and showed the gene expression signatures of central memory T cells (Tcm) (C3), effector memory T cells (Tem) (C3), and tissue-resident memory T cells (Trm) (C2 and C3). The CD8_TCR135pos_C2 cluster also showed gene expression signatures of exhausted T cells (Tex). Cluster CD8_TCR135pos_C4 were proliferating cells (Tprol). Similar to scRNA-Seq data, the FACS analysis also revealed that tumor-infiltrating TCR135-T cells had increased levels of exhaustion markers (TIM3 and PD-1) and tissue-resident memory markers (CD69 and CD103) (Supplemental Figure 8). These findings reveal the memory and exhausted gene expression features in tumor-infiltrated TCR135pos T cells, suggesting that these T cells had been experiencing antigen stimulation.

### TCR135 specifically recognized APC-presented EBNA1 and mediated the indirect killing of HLA II–negative tumor cells in a TNF-α–dependent manner.

**Figure 7:**
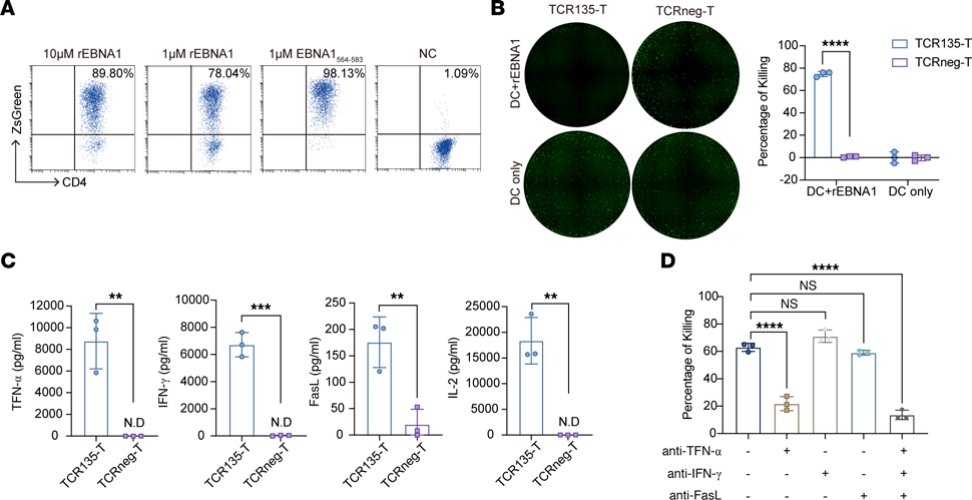
TCR135 specifically recognized EBNA1 presented by APCs and mediated indirect killing of HLA II–negative tumor cells in a TNF-α–dependent manner. (**A**) TCR135-transduced JK4NF cells were cocultured overnight with DCs pulsed with 10 μM or 1 μM GST–C-terminus EBNA1 recombinant protein (rEBNA1), 1 μM EBNA1_564–583_ peptide, or PBS (NC). The frequency of ZsGreen-expressing cells was measured by flow cytometry. (**B**) SNU-719-EBNA1 tumor cells were cocultured with DCs pulsed with rEBNA1 protein (DC + rEBNA1) or PBS (DC only) and TCR135-transduced T cells (TCR135-T) or non-transduced T cells (TCRneg-T) for 72 hours, and then the living tumor cells were analyzed by Celigo Image Cytometer fluorescence photography. The representative fluorescent images and statistical results are shown. 2-way ANOVA and Šidák’s multiple comparisons, ****P < 0.0001. (**C**) DCs pulsed with rEBNA1 protein were cocultured with TCR135-T cells or TCRneg-T cells for 24 hours. Afterward, the supernatant was collected, and the cytotoxic effectors were detected using RayPlex Human Cytotoxic T Cell Array Kit 1. 2-tailed unpaired Student’s *t* test, **P < 0.01, ***P < 0.001. ND, not detected. (**D**) DCs pulsed with rEBNA1 protein were cocultured with TCR135-T cells for 48 hours. Then, the supernatant was added to SNU-719-EBNA1 tumor cells and cultured for 72 hours, with or without neutralizing antibodies against TNF-α, IFN-γ, and FasL. The analysis of living tumor cells was carried out using Celigo Image Cytometer fluorescence photography. 1-way ANOVA and Dunnett’s multiple comparisons, ****P < 0.0001.

CD4^+^ T cells may kill HLA II–negative tumors when tumor antigen is processed and presented by antigen-presenting cells (APCs) (11, 38). Thus, we explored whether TCR135 could mediate the indirect killing of HLA II–negative tumor cells. First, the GST-tagged C-terminus EBNA1 recombinant protein (rEBNA1) was purified and added to the culture medium of HLA-DP5^+^ dendritic cells (DCs). TCR135-transduced JK4NF cells were activated by DCs preloaded with rEBNA1 (Figure 7A). Furthermore, we used HLA-DP5^+^ or HLA-DP5^–^ PBMCs to analyze whether this recognition was HLA-DP5 dependent. Indeed, only HLA-DP5^+^ PBMCs preloaded with rEBNA1 successfully activated TCR135 (Supplemental Figure 9A), suggesting that TCR135 can specifically recognize EBNA1 presented by HLA-DP5^+^ APCs. To further mimic the in vivo conditions, the cell lysates of HLA II–positive 293T-CIITA-DP5-EBNA1 or HLA II–negative 293T-EBNA1 were loaded onto HLA-DP5^+^ or HLA-DP5^–^ PBMCs. DP5^+^ PBMCs activated TCR135 after exposure to both HLA-positive and HLA-negative cell lysates (Supplemental Figure 9, B and C). To further investigate the ability of TCR135-T cells to kill HLA II–negative tumor cells indirectly, TCR135-T cells were cocultured with HLA II–negative SNU-719-EBNA1 tumor cells, together with autologous DCs preloaded with rEBNA1 protein or DCs alone, as a control. TCR135-T cells significantly inhibited the growth of HLA II–negative SNU-719-EBNA1 tumor cells when cocultured with rEBNA1-loaded DCs (Figure 7B). The cytotoxic effectors released in the coculturing supernatant of T cells and rEBNA1-loaded DCs were then quantified. After coculturing with rEBNA1-loaded DCs, TCR135-T cells secreted significantly more TNF-α, IFN-γ, FasL, and IL-2 compared with TCRneg-T cells, as shown in Figure 7C and Supplemental Figure 10. TCR135-T cell and rEBNA1-loaded DC coculture supernatant was sufficient to kill SNU-719-EBNA1 tumor cells without the need for cell-cell contact, as shown in Figure 7D. Interestingly, cytolysis of SNU-719-EBNA1 tumor cells by the coculturing supernatant was significantly inhibited when a TNF-α–neutralizing antibody was added (Figure 7D). These findings indicate that TCR135 can not only directly kill HLA-DP5^+^EBNA1^+^ tumor cells, but also recognize EBNA1 presented by APCs and then mediate indirect killing of HLA II–negative tumor cells in a TNF-α–dependent manner.

### HLA-DP and EBNA1 expression on NPC and gastric cancer cells.

**Figure 8:**
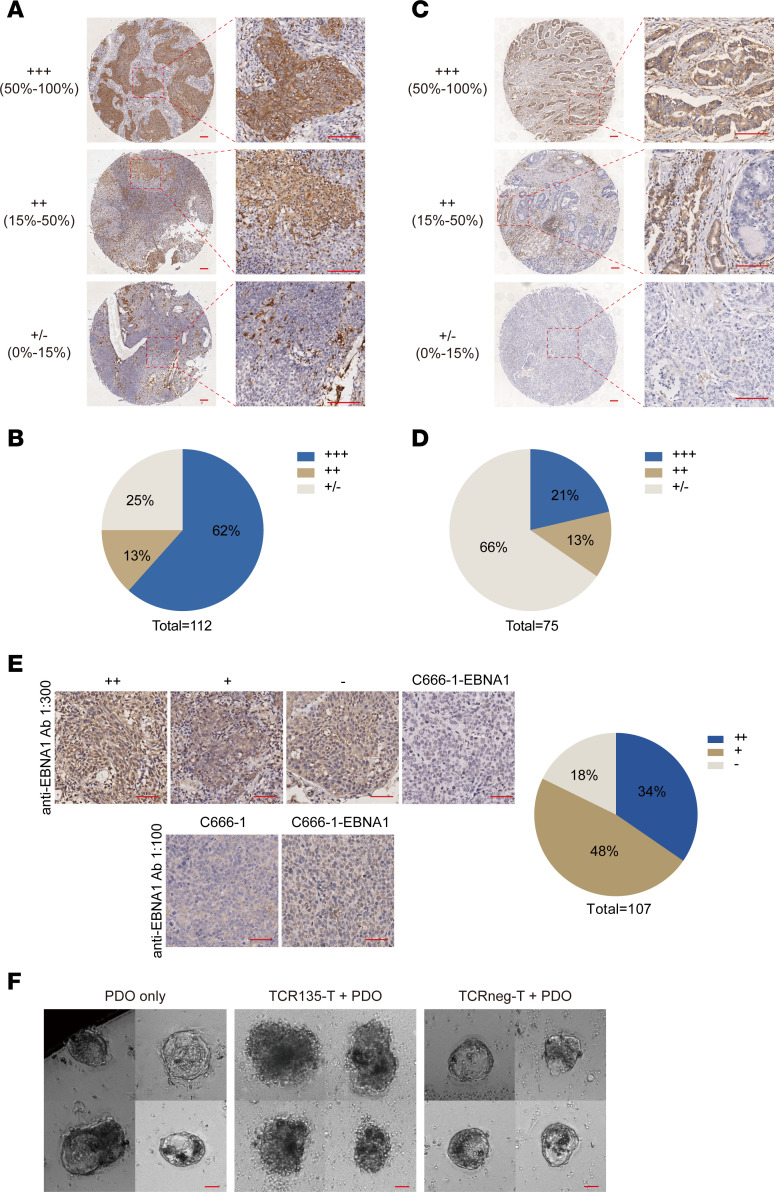
HLA-DP and EBNA1 expression on NPC and gastric cancer cells. (**A** and **B**) The tumor tissue microarray of NPC (*n* = 112) was stained with anti–HLA-DPB1 antibody by immunohistochemistry and was scored by the proportion of HLA-DP^+^ tumor cells in total tumor cells (**A**). The quantitative result is shown in the pie chart (**B**). Scale bars: 100 μm. (**C** and **D**) The tumor tissue microarray of gastric cancer (*n* = 75) was stained with anti–HLA-DPB1 antibody by immunohistochemistry and was scored by the proportion of HLA-DP^+^ tumor cells in total tumor cells (**C**). The quantitative result is shown in the pie chart (**D**). Scale bars: 100 μm. (**E**) The tumor tissue sections of C666-1 or C666-1-EBNA1 mouse xenograft and the tumor tissue microarray of NPC patients were stained using an anti-EBNA1 antibody. The staining intensity of EBNA1 in the tumor tissue microarray of NPC patients was scored, and the quantitative results (*n* = 107) are shown in the pie chart. Scale bars: 100 μm. (**F**) TCR135-transduced T cells (TCR135-T) or non-transduced T cells (TCRneg-T) were cocultured with PDOs for 48 hours, and then bright-field images were captured. The representative microscopy images are shown. Scale bars: 50 μm.

To further investigate the therapeutic implications of TCR135 in the clinic, we explored the expression of HLA-DP molecules on NPC and gastric cancer cells. First, the tumor tissue microarray of 112 patients with NPC was stained with anti–HLA-DPB1 antibody by immunohistochemistry, and the expression of HLA-DP on tumor cells was quantified (Figure 8A). Surprisingly, we found that 62% (69 of 112) of NPC patients showed strong expression of HLA-DP on tumor cells and 13% of cases showed medium expression, while only 25% of cases had almost no expression of HLA-DP on tumor cells (Figure 8B). We then assessed the expression of HLA-DP in the tumor tissue microarray of 75 patients with gastric cancer. The expression of HLA-DP in gastric cancer was lower, with only 21% of patients showing 50%–100% expression of HLA-DP on tumor cells and 66% of cases having almost no expression of HLA-DP on tumor cells (Figure 8, C and D).

We evaluated the expression level of EBNA1 in NPC tumor cells using immunohistochemistry. Tumor tissue sections of C666-1 or C666-1-EBNA1 mouse xenograft and tumor tissue microarray of NPC patients were stained with anti-EBNA1 antibody. As shown in Figure 8E, nuclear staining of EBNA1 was detected in both the C666-1-EBNA1 tumor section and NPC tumor tissue microarray, but not in the C666-1 tumor section. Additionally, the staining signal was stronger in NPC tumor cells compared with C666-1-EBNA1 cells. These results indicate that the endogenous expression level of EBNA1 in NPC tumor cells was higher than that in the constructed C666-1-EBNA1 cells. We quantified the expression of EBNA1 in NPC tumor tissue microarray and found that approximately 80% of NPC patients (88 of 107) exhibited positive expression of EBNA1 in tumor cells (++, 34%; +, 48%) (Figure 8E).

In addition, we used patient-derived organoids (PDOs) to evaluate the activity of TCR135-transduced T cells toward primary NPCs. One of the PDOs was found to carry HLA-DPB1*05:01/DPA1*02:02 genotype and express both EBNA1 and HLA-DP molecules (Supplemental Figure 11). We observed that TCR135-transduced T cells were capable of killing this particular PDO. As shown in Figure 8F, TCR135-T cell–treated PDOs displayed destroyed PDO structures, while TCRneg-T cell–treated PDOs remained unaltered with intact cystic PDO appearance. Together, these findings suggest that TCR135 holds great potential as a candidate for EBV-related NPC immunotherapy.

## Discussion

In this study, we screened for CD4 TCRs that recognized EBNA1 presented by HLA-DP5 (HLA-DPA1*0202/DPB1*0501), one of the most common genotypes of HLA class II molecules in East Asia and Oceania (39). The EBNA1_567–581_ peptide naturally processed by HLA-DP5 was identified by MS for the first time to our knowledge in this study. We identified TCR135, a CD4 TCR specific to EBNA1_567–581_ with high functional avidity. TCR135-transduced CD4^+^ T cells showed strong Th1-cytokine production in an HLA-DP5–restricted, EBNA1_567–581_-specific manner. CD8^+^ T cells were also activated by TCR135 and recognized EBNA1_567–581_. In addition, TCR135-transduced T cells were found to directly kill HLA-DP5^+^EBNA1^+^ tumor cells and recognize EBNA1 presented by DCs, thereby killing HLA-DP5–negative tumor cells. Moreover, TCR135-transduced T cells significantly inhibited tumor growth in vivo in xenograft nasopharyngeal tumor models. TCR135 identified in this study may provide a new strategy for EBV-related nasopharyngeal cancer immunotherapy in HLA-DP5^+^ patients.

To date, most TCR-T investigations have focused on CD8^+^ T cells. The downregulation or loss of HLA class I molecules can prevent tumor cells from being recognized by CD8^+^ cytotoxic T cells. HLA downregulation is common in cancers (40), with the percentage of total or partial HLA loss ranging from 0% to 93% depending on the type of cancer (41). Impaired HLA class I antigen processing and presentation is thus one mechanism of acquired resistance to immune checkpoint therapy (42, 43). A similar phenomenon is also found in T cell therapy for various cancers. In a patient with metastatic colon cancer receiving HLA-C*08:02–restricted CD8^+^ T cell therapy, 1 of 7 lesions that had progressed 9 months after therapy was found to have lost the chromosome 6 haplotype encoding the HLA-C*08:02 molecule (44). In another patient with chemo-refractory breast cancer treated with an HLA-A*02–restricted TCR specific for the p53R175H mutation, the vast majority of the tumor cells in the progressing lesion did not express HLA-A*02:01, although high expression of HLA-A*02:01 was observed in the tumor cells during the pretreatment biopsy (45). In the phase I/II clinical trial of CD8 TCR-T for HPV E6, 2 patients experienced treatment failure, one of which was due to the loss of HLA class I alleles (46). Similarly, the loss of the HLA class I pathway caused by HLA or β_2_-microglobulin mutation was observed in tumors with primary or secondary resistance to CD8 TCR-T therapy targeting HPV E7 (47). CD4^+^ T cells can kill tumor cells through HLA class II molecules presented by tumor cells or APCs (48). Therefore, we propose that CD4^+^ T cell therapy may prevent acquired tumor resistance caused by impaired HLA class I antigen processing and presentation.

The combination of CD4^+^ and CD8^+^ T cell therapy may be the best choice. Mouse models combining CD4^+^ and CD8^+^ T cells for adoptive transfer can induce bystander killing of antigen-negative tumor cells, which may prevent acquired tumor resistance caused by downregulation or deletion of the targeted tumor antigens (49). Poncette et al. (11) identified an HLA-DRA/DRB1*0401–restricted NY-ESO-1–specific CD4 TCR that caused tumor regression in combination with NY-ESO-1–redirected CD8^+^ T cells in a mouse model of adoptive T cell therapy. They used DR4×Rag^–/–^ mice inoculated with HLA-DR4–negative tumor cells in which the CD8 epitope was only present on the tumor cells and the CD4 epitope was only present on the host cells. The combination of NY-ESO-1–specific CD4^+^ and CD8^+^ T cells showed significantly better efficacy than either CD4^+^ or CD8^+^ T cells on their own. We also show that TCR135-transduced T cells recognized EBNA1 presented by APCs and killed HLA-DP5–negative tumor cells in vitro (Figure 6), which suggests a role for TCR135-transduced T cells in bystander killing of antigen-negative tumor cells. Unfortunately, we did not have HLA-DP5–transgenic mice. We are planning to investigate the cooperation of EBNA1-specific CD4^+^ and CD8^+^ T cells in a future study.

To the best of our knowledge, this is the first time that the expression of HLA-DP molecules has been analyzed in NPC cells. The majority of NPC patients showed 50%–100% expression of HLA-DP on tumor cells, indicating that NPC is well suited for CD4 TCR-T therapy. The expression of HLA-DP in gastric cancer was relatively low, with only one-fifth as many patients showing 50%–100% expression of HLA-DP on tumor cells. We wondered whether the lower expression of HLA-DP in gastric cancer was because of the poor prevalence of EBV among gastric cancer patients since EBV-associated gastric cancer comprises less than 10% of all gastric carcinomas worldwide (50). Thus, we then assessed the expression of HLA-DP on paraffin-embedded tissues from 13 EBV-positive and 30 EBV-negative gastric cancer patients. However, the tumor cells of EBV-positive and -negative gastric cancer showed similar expression patterns of HLA-DP, with about 30% of cases showing strong expression (Supplemental Figure 12). Ghasemi et al. (51) have reported that HLA class II genes are significantly upregulated in EBV-associated gastric cancer compared with normal tissues and other gastric cancer subtypes. Since they used RNA sequencing data from whole tumor tissue with both tumor cells and infiltrated immune cells, we believe the upregulation of HLA class II genes was mainly attributable to the elevated immune cell infiltration in EBV-associated gastric cancer.

TCR135-T therapy has the potential to benefit patients with any type of cancer who are HLA-DP5^+^EBNA1^+^. Typically, TCR-T clinical trials are basket trials, meaning they are not specific to any one kind of tumor. For example, the phase I trial of MAGE-A4–specific TCR-T cells has included patients with synovial sarcoma, ovarian cancer, and head and neck cancer (52). NY-ESO-1–specific TCR-T cells have also been tested in phase I trials in patients with melanoma (53), synovial sarcoma (54), and multiple myeloma (55). In our study, we have found that the NPC cell line C666-1 and the gastric cell line SNU-719 can be targeted by TCR135-T cells (Figure 2A and Figure 7, B and D). It will be interesting to further investigate whether other EBV-related tumor types such as B cell lymphoma and extranodal NK/T cell lymphoma can be targeted by TCR135-T cells.

The naturally processed EBNA_567–581_ peptide was first identified by MS in this study. HLA class II molecules usually bind peptides 12–25 residues in length according to their open binding grooves. The EBNA_564–583_ 20-mer peptide has been used in analyzing specific CD4^+^ T cell response in HLA-DP5^+^ EBV-seropositive healthy donors (24) and for vaccinating EBV-associated NPC patients (56). However, the peptide within EBNA_564–583_ that is naturally processed by the HLA-DP5 molecule has, to our knowledge, not been investigated before. In this study, we detected the presence of the EBNA_567–581_ 15-mer peptide but not the EBNA_564–583_ 20-mer peptide in the eluted HLA-DP5 immunopeptidome by LC-MS/MS (Figure 2). In addition, we showed that the EBNA_567–581_ peptide could only be endogenously presented by HLA-DPA1*02:02/DPB1*0501 but not by HLA-DPA1*01:03/DPB1*0501, which indicates that the α chain could also influence the HLA-DP–presented peptidome.

The phenotypic analysis of tumor-infiltrated TCR135-transduced T cells showed that TCR135-positive CD4^+^ and CD8^+^ T cells both exhibited the tissue-resident memory gene expression feature in the tumor microenvironment. Many groups have found that neoantigen-specific CD4^+^ T cells in the tumor microenvironment of various cancer types express *CXCL13* (57–60). *ZNF683* was also a marker for tumor-specific tissue-resident memory CD8^+^ T cells (58, 61, 62). In our study, we found that *CXCL13* was specifically upregulated in tumor-infiltrated TCR135-positive CD4^+^ T cells, while *ZNF683* was specifically upregulated in tumor-infiltrated TCR135-positive CD8^+^ T cells (Supplemental Figure 13). These results suggest that tumor-infiltrated TCR135-transduced T cells displayed similar gene signatures to tumor-specific T cells in the clinic. Tumor-infiltrated TCR135-transduced T cells also exhibited increased expression of exhaustion markers. TIM3 and PD-1, which are commonly referred to as exhaustion markers, can also be upregulated in activated cells. Because the tumor-infiltrated TCR135-transduced T cells were harvested at an early time point (day 7), TIM3 and PD-1 overexpression first suggested that these T cells had been subjected to antigen stimulation. It is still possible that these T cells will become dysfunctional after repeated antigen exposure. Thus, it may be necessary to combine CD4 TCR-T therapy with checkpoint inhibitor therapy in the clinic. Additionally, a small cluster of TCR135-transduced CD4^+^ T cells differentiated into Tregs within the tumor microenvironment. Investigating the function of these cells during the antitumor response of TCR-T therapy would be an intriguing avenue for future research.

## Methods

Further information can be found in Supplemental Methods.

### Sex as a biological variable.

Sex was not considered as a biological variable in this study.

### Cell line.

Cell lines were cultured in RPMI 1640 (J.RT3-T3.5, C666-1, and SNU-719) or DMEM (293T) supplemented with 10% FBS (Excell) and 100 U/mL penicillin/streptomycin (Life Technologies). The J.RT3-T3.5 cell line was purchased from ATCC, and the C666-1 and SNU-719 cell lines were purchased from CTCC. C666-1 is an EBV^+^ and HLA-DP5^+^ NPC cell line. SNU-719 is an EBV^+^ gastric cancer cell line. All cell lines used in the experiments were mycoplasma-free.

The J.RT3-T3.5 cell line was used to generate the JK4NF reporter cell line by single-cell cloning as described before (63). The genes encoding CIITA, HLA-DPA1*02:02/DPB1*0501, HLA-DPA1*01:03/DPB1*0501, full-length EBNA1, and C-terminus EBNA1_473–616_ were synthesized, and constructed into the pLKO.1puro (Addgene 8453) lentiviral vectors under EF-1α promoter. P2A-linked GFP/mCherry was used as the reporter. The target cell lines were constructed by serial transduction with corresponding lentiviral vectors and sorted by flow cytometry.

### Generation of EBNA1_564–583_-specific T cells.

EBNA1_564–583_-specific T cells were enriched from HLA-DP5^+^ healthy donors as described before (63). Briefly, PBMCs were isolated by density-gradient centrifugation from buffy coats (Guangzhou Blood Center) and cryopreserved. CD14^+^ cells were isolated from PBMCs using positive selection with magnetic beads (Stemcell Technologies) according to the manufacturer’s instructions. Purified CD14^+^ cells were cultured in RPMI 1640 medium containing 10% FBS, 800 IU/mL GM-CSF, and 500 IU/mL IL-4. On day 3, fresh medium containing 1,600 IU/mL GM-CSF and 1,000 IU/mL IL-4 was added. To generate mature DCs, IL-1β, IL-6, TNF-α, and PGE2 were added at concentrations of 10 ng/mL, 10 ng/mL, 10 ng/mL, and 1 μg/mL, respectively, for 48 hours, starting from day 5.

CD4^+^ naive T cells were enriched from autologous CD14^–^ cells by negative selection (Stemcell Technologies). Mature DCs were loaded with 40 μg/mL peptide (EBNA1_564–583_) at 37°C for 2 hours. Isolated CD4^+^ naive T cells (2 × 10^6^ cells) and peptide-loaded DCs (2 × 10^5^ cells) were cocultured at a ratio of 10:1 in TexMACS medium supplemented with 10% Human Serum AB (GeminiBio) and 60 ng/mL of IL-21 in 6-well plates. Then 10 IU/mL IL-2, 10 ng/mL IL-7, and 10 ng/mL IL-15 were added every other day. After 7 days, T cells were restimulated with peptide-loaded DCs. After 14 days, T cells were stained with allophycocyanin-tetramer and FITC-CD4 and analyzed by flow cytometry.

### TCR sequencing and cloning.

Single CD4^+^tetramer^+^ T cells were sorted into 96-well plates and amplified using single-cell PCR to obtain TCR sequences. TCR sequences from the sorted single cells were obtained by a series of 2 nested PCR reactions as previously described (64). TCR sequences were analyzed using the IMGT/V-Quest tool (https://www.imgt.org/). TCR α/β chains were synthesized by GenScript and cloned into the lentiviral vector pLKO (63). The human TCR constant regions were replaced with mouse TCR constant regions to ensure a preferred pairing of transgenic TCR chains (65, 66).

### Lentiviral production.

Lentiviral particles used in this experiment were produced by transient transfection of 293T cells with target vector plasmid and packaging plasmids (Addgene) encoding pMD2G, REV, and RRE and concentrated with a 50 kDa ultrafiltration tube before storage at –80°C.

### Generation of EBNA1_564–583_-specific TCR gene-transduced JK4NF cells and T cells.

JK4NF cells were transduced with lentiviral particles at a multiplicity of infection (MOI) of 10 to construct JK4NF-TCR cell lines.

Thawed PBMCs (2 × 10^6^ cells/mL) or isolated CD4^+^ or CD8^+^ T cells (1 × 10^6^ cells/mL) were activated with human T-activator CD3/CD28 (Miltenyi Biotec) at recommended titer of 1:100. After 24 hours, T cells were twice transduced with lentiviral particles at an MOI of 1 in the presence of protamine (10 μg/mL). TCR-transduced T cells were then expanded in RPMI 1640 medium supplemented with 10% FBS (Gibco), 1% penicillin/streptomycin, and 200 IU/mL IL-2. The transduction efficiency was calculated by staining for mouse TCRβ and tetramer.

### In vitro functional assay.

For T cell activation assays, JK4NF-TCR cells were cocultured with tumor cells and loaded with or without EBNA1_564–583_ peptide, at an effector/target (E/T) ratio of 1:1. After 16 hours of incubation at 37°C, cells were analyzed by flow cytometry to determine the percentage of ZsGreen^+^ JK4NF-TCR cells for each sample. EC_50_ values were estimated using a nonlinear regression curve and fit with a variable slope (4 parameters) in Prism 8 (GraphPad Software).

For ELISA assays, 2,000 TCR-transduced T cells were cocultured with 20,000 peptide-pulsed 293T-DP5-CIITA cells. Coculture supernatants were harvested after 18–24 hours, and IFN-γ concentration was measured by ELISA (Invitrogen 88-7316-88). For tumor cell lines, 20,000 TCR-transduced T cells were cocultured with an equal number of tumor cells for 24 hours, and IFN-γ concentration was measured by ELISA (Invitrogen 88-7316-88).

For cytokine production assays, 50,000 mTRBC^+^ T cells were cocultured with 50,000 target cells in the presence of GolgiPlug and GolgiStop (BD Biosciences) at recommended concentrations. To verify the restriction of HLA-DP5, additional HLA-DP antibody clone B7/21 or PBS was added at 1:100 from the start of the coculture. For CD154 assays, Alexa Fluor 700–anti–human CD154 antibody was added from the start of the coincubation. After 12 hours of incubation, cells were intracellularly stained using the Cytofix/Cytoperm Fixation/Permeabilization Kit (BD Biosciences), according to the manufacturer’s instructions.

Target cell lysis was evaluated with the Celigo Imaging Cytometer (Nexcelom Bioscience). TCR-transduced T cells were cocultured with target cells at the indicated E/T ratio in 96-well plates for 72 hours. To perform Celigo image cytometry, sample plates with cells were loaded into the instrument, using preset scan and analysis settings. For tumor cells, Celigo was set up to acquire images in the Green fluorescence channel, and the exposure times were 100,000 microseconds. Each well was captured with 16 photographs by Celigo, which were then assembled into a comprehensive image for analyzing cell confluence. Results were exported as the ratio of fluorescent cell area to the area of the well (Confluence%). The percentage of killings is shown in the figures.
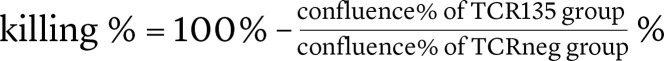


### In vivo mouse xenograft models.

Female NOD/SCID/IL2Rg^–/–^ (NCG) mice aged 3–4 weeks were purchased from GemPharmatech. All experimental procedures were approved by the Guangzhou Medical University Institutional Animal Care and Use Committee. NCG mice were injected subcutaneously with 5 × 10^6^ C666-1-EBNA1 tumor cells into one flank. When tumor volume reached 80–100 mm^3^, 1 × 10^7^ TCR135-transduced T cells or non-transduced T cells were administered by retro-orbital injection. At the same time, 50,000 IU IL-2 was administered intraperitoneally, and this was repeated every 24 hours for 5 days. Tumor size was measured every 2 days with calipers in 2 perpendicular directions, and tumor volumes were calculated using the formula: volume (mm^3^) = (length × width^2^)/2. When tumor volume reached 2,000 mm^3^, mice were sacrificed, and tumors were dissected out.

To analyze T cell infiltration, peripheral blood, spleen, and tumor samples were collected 7 days after T cell injection. The tumor tissues were dissociated into single-cell suspension by enzyme digestion (MilliporeSigma). The single-cell suspension was stained with PE/Cyanine7–anti–human CD45 (BD Biosciences), allophycocyanin–TCR β chain, Pacific blue–CD8, and PE-CD4, and sorted by FACS based on CD45. These CD45^+^ cells were used for scRNA-Seq.

### Indirect killing assay.

Monocytes were isolated from PBMCs using positive selection with magnetic beads coated with antibodies recognizing CD14 (BioLegend) according to the manufacturer’s instructions. CD14^+^ cells were cultured for 5 days in RPMI 1640 medium supplemented with 10% FBS (Gibco), 1% penicillin/streptomycin, 800 IU/mL of GM-CSF, and 500 IU/mL of IL-4. The immature DCs were loaded with rEBNA1 protein at 37°C for 5 hours, and then 10 ng/mL IL-1β, 10 ng/mL IL-6, 10 ng/mL TNF-α, and 1 μg/mL PGE2 were added to induce DC maturation for 12 hours. JK4NF cells (6 × 10^4^ cells) were cocultured with the mature DCs (6 × 10^4^ cells) loaded with or without rEBNA1 protein at a ratio of 1:1 overnight and then analyzed by flow cytometry to determine the percentage of ZsGreen^+^ cells. TCR135-T cells or TCRneg-T cells (6 × 10^4^ cells) were cocultured with HLA II–negative SNU-719-EBNA1 tumor cells (2 × 10^4^ cells), along with autologous DCs (1.2 × 10^4^ cells). The DCs were either preloaded with rEBNA1 protein or used alone in 96-well plates. After 72 hours, cell confluence was measured using Celigo. Each well was captured with 16 photographs, which were then assembled into a comprehensive image for analyzing cell confluence. The results were exported as the ratio of fluorescent cell area to the area of the whole well (Confluence%). The percentage of killing was calculated as follows:


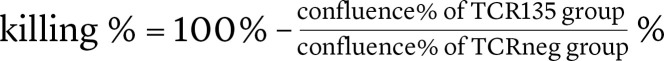


In some experiments, TCR135-T cells or TCRneg-T cells were cocultured with the mature DCs loaded with rEBNA1 protein at an E/T ratio of 5:1. The supernatant was collected, and the concentrations of 12 cytokines in the supernatant were quantitatively measured using RayPlex Human Cytotoxic T Cell Array Kit 1 (Ray Biotech) and flow cytometry. HLA II–negative SNU-719-EBNA1 tumor cells were then cultured with these supernatants, with or without neutralizing antibodies against TNF-α (Cell Signaling Technology, clone D1B4; 1 μg/mL), IFN-γ (BioLegend, clone W19171A; 10 μg/mL), and FasL (BioLegend, clone W19403O; 1 μg/mL).

### Patient-derived organoid models.

For NPC organoid models, organoids were established from NPC tumor tissues by Accurate International Biotechnology Co. as described before (67). HLA typing of organoids was done by Bfrbiotech Co. using PCR sequencing–based typing. The expression of EBNA1 and HLA-DPB1 was analyzed by immunohistochemistry. The EBNA1^+^HLA-DP5^+^ PDO was selected for the following coculture assay. PDO cells were cocultured with TCR135-T cells or TCRneg-T cells in 96-well plates. After 48 hours, microscopy images were captured with a Biotek Cytation 5 cell imaging multimode reader.

### Statistics.

Differences between the 2 groups were compared using a 2-tailed unpaired Student’s *t* test conducted using Prism 8 software (GraphPad Software). Differences between multiple groups were compared using 1-way or 2-way ANOVA, followed by Šidák’s or Dunnett’s multiple-comparison post-test. A 2-way ANOVA test was used to compare the tumor growth curves between TCR135-T and TCRneg-T cell–treated groups. All data are expressed as mean ± SD. *P* values less than 0.05 were considered statistically significant.

### Study approval.

All animal handling and tumor experiments were approved by the Guangzhou Medical University Institutional Animal Care and Use Committee (reference GY2022-040). The use of healthy donor buffy coats was approved by the ethics committee of Guangzhou Blood Center. The use of tumor tissue microarray of NPC and gastric cancer patients was approved by the ethics committee of Shanghai Outdo Biotech Co. The use of tumor tissue of NPC for organoid culture was approved by the ethics committee of Nanfang Hospital, Southern Medical University.

### Data availability.

RNA-Seq data are available at Genome Sequence Archive (accession no. PRJCA016403). Values for all data points in graphs are reported in the Supporting Data Values file.

## Author contributions

Lin Chen, ZW, HL, and XW designed the study. CW, JC, JL, ZX, LH, XL, CX, and BW performed the experiments and collected data. QZ and Lei Chen performed the LC-MS/MS and collected data. HY and HH performed the immunohistochemistry staining and the pathological analysis. GL and DD collected the NPC tumor tissue for organoid culture. Lin Chen, ZW, CW, JC, and JL wrote the manuscript. The order among co–first authors was assigned based on contributions.

## Supplementary Material

Supplemental data

Supplemental tables 1-2

Supporting data values

## Version 1 02/27/2024

In-Press Preview

## Version 2 04/15/2024

Electronic publication
