# Supplementary material for: An EBV-related CD4 TCR immunotherapy inhibits tumor growth in an HLA-DP5+ nasopharyngeal cancer mouse model
Source: J Clin Invest. 2024 Feb 27;134(8):e172092. doi: 10.1172/JCI172092 (PMC11014665; doi:10.1172/JCI172092)
Supplement: Supplemental data [file jci-134-172092-s050.pdf]

Supplementary Figures

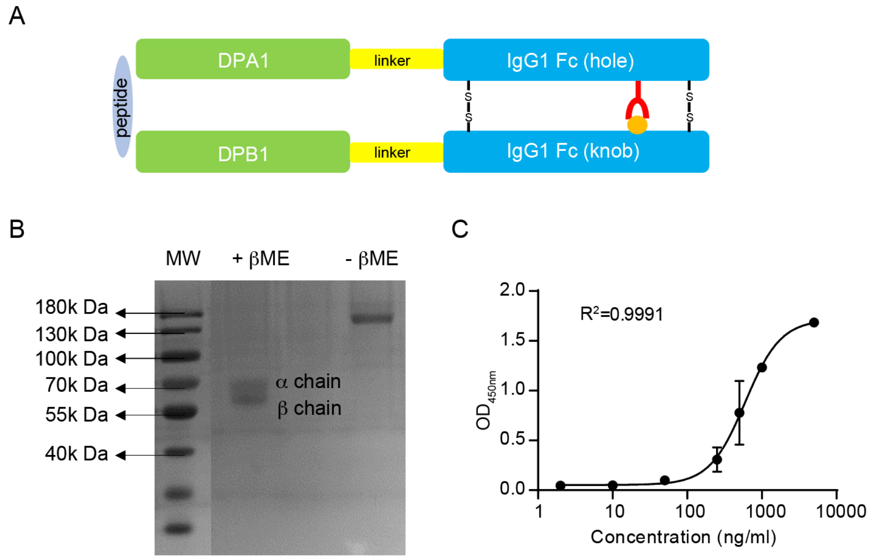

Supplementary Figure 1. Production of empty HLA-DP5 monomer. (A) Schematic for HLA-DP5 monomer construction. **(B)** SDS-PAGE assays for the detection of HLA-DP5 monomer. +βME or -βME means the loading buffer containing βME or not. **(C)** ELISA assays for the analysis of the titrated concentration of HLA-DP5 monomer.

A

Cell line	DPA1*	DPB1*
293T	01:03<br>01:03	04:02<br>04:02
SNU-719	01:03<br>02:02	04:02<br>05:01
C666-1	02:02<br>02:02	05:01P<br>05:01P

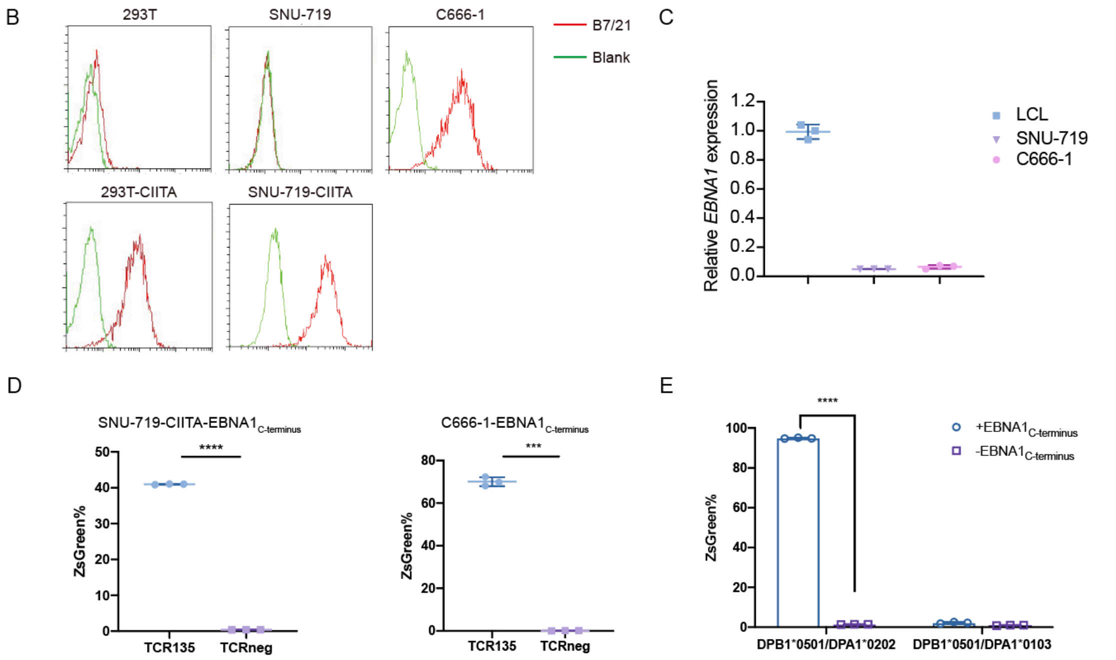

Supplementary Figure 2. EBNA1$_{564\text{-}583}$ could be endogenously processed by HLA-DPA1*02:02/DPB1*0501. (A) HLA-DP genotype of indicated cell lines. **(B)** The expression of HLA-DP on the cell surface of indicated cell lines analyzed by flow cytometry. **(C)** Quantitative Real-time PCR (qPCR) assays for the relative *EBNA1* expression in LCL, SNU-719, and C666-1. **(D)** The percentage of ZsGreen expression in TCR135-transduced (TCR135) or non-transduced (TCRneg) JK4NF cells after coculture with SNU-719-CIITA-EBNA1$_{C\text{-}Terminus}$ and C666-1-EBNA1$_{C\text{-}Terminus}$ cells. **(E)** The percentage of ZsGreen expression in TCR135-transduced JK4NF cells after coculture with 293T-CIITA-DPA1*0202/DPB1*0501 or 293T-CIITA-DPA1*0103/DPB1*0501 transduced with (+EBNA1$_{C\text{-}Terminus}$) or without (-EBNA1$_{C\text{-}Terminus}$) C-Terminus EBNA1. Two-way ANOVA and Sidak's multiple comparisons, ****, $P < 0.0001$. The 293T-CIITA-DPA1*0202/DPB1*0501 or 293T-CIITA-DPA1*0103/DPB1*0501 without EBNA1/C-Terminus EBNA1 groups were shared between Figure 2C and supplementary Figure 2E.

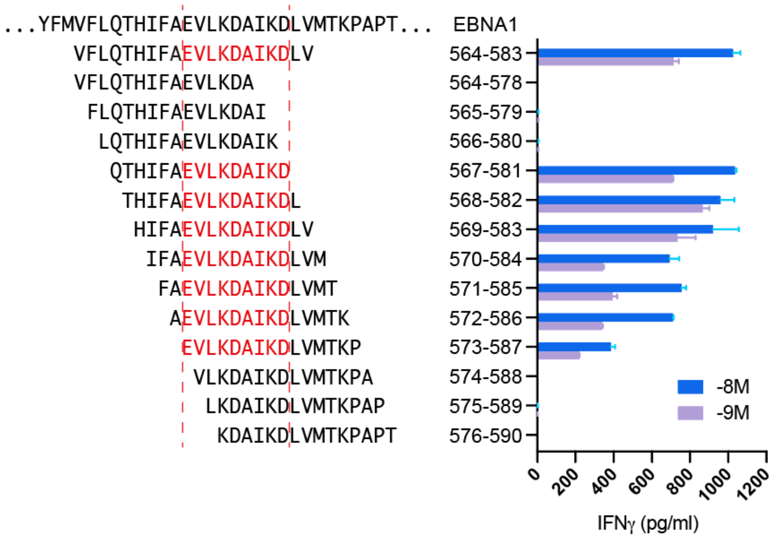

Supplementary Figure 3. EBNA1$_{573\text{-}581}$ was the minimal epitope that could activate TCR135 transduced CD4^{+} T cells. The right panel shows the concentration of IFNγ released by TCR135-transduced CD4^{+} T cells cocultured with indicated overlapping peptides pulsed 293T-CIITA-DP5. The left panel shows the amino acid sequences of overlapping peptides. The red fonts show the minimal epitope EBNA1$_{573\text{-}581}$ recognized by TCR135.

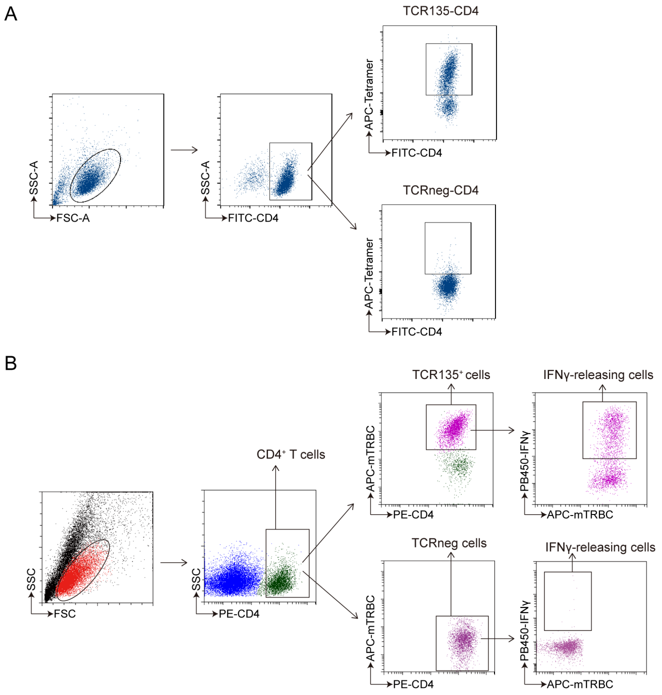

Supplementary Figure 4. Gating strategy for tetramer staining and intracellular cytokine staining of TCR135-transduced cells and non-transduced control cells.

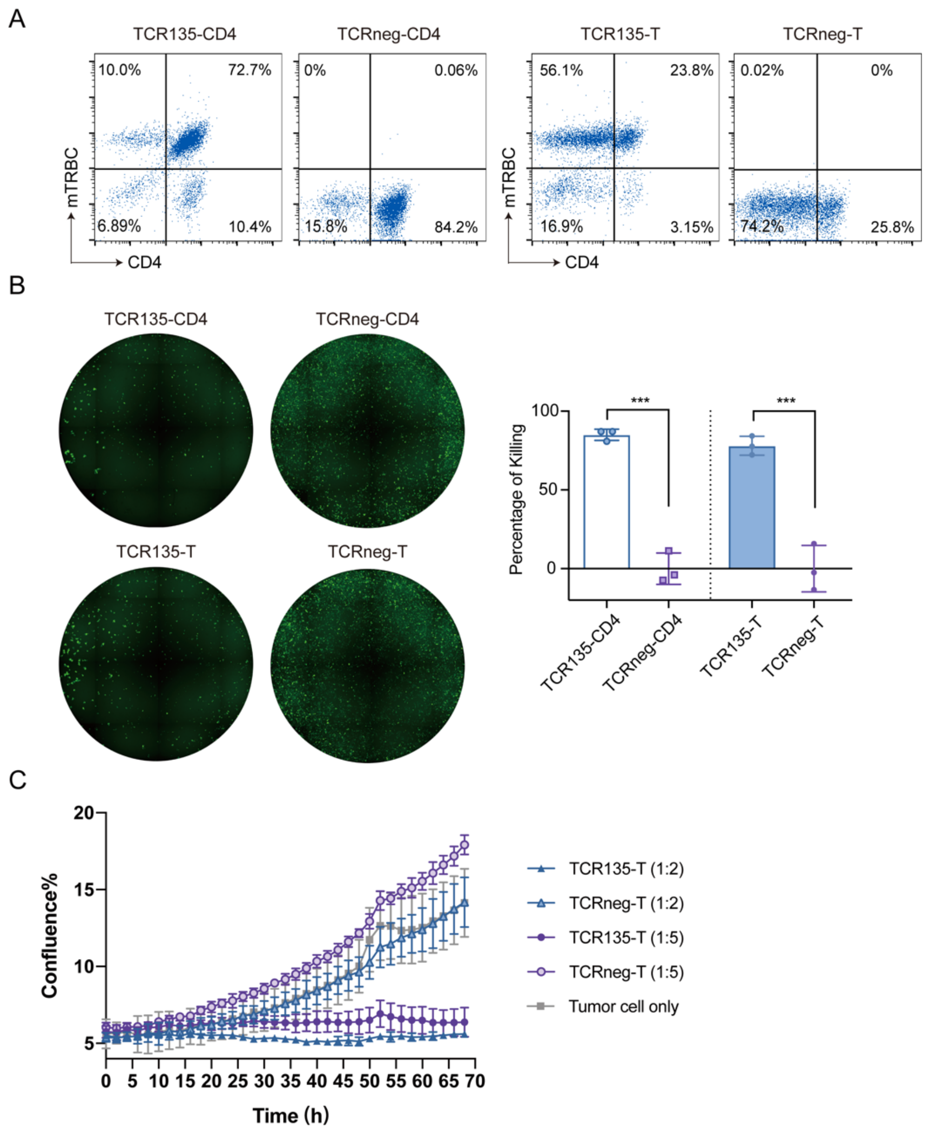

Supplementary Figure 5. TCR135 transduced T cells successfully inhibited the growth of C666-1-EBNA1 tumor cells. (A) Flow cytometry analysis of the expression of TCR135 on TCR135-transduced (TCR135-CD4) or non-transduced (TCRneg-CD4) primary CD4 T cells, and TCR135-transduced (TCR135-T) or non-transduced (TCRneg-T) PBMCs. T cells were stained with anti-mouse TCRβ (mTRBC) and anti-human CD4 (CD4). **(B)** The TCR135 transduced $CD4^+$ T cells (TCR135-CD4) or PBMCs (TCR135-T) were cocultured with C666-1-EBNA1 cells at an E/T ratio of 1:2 for 72 hours, then the living tumor cells were analyzed by Celigo Image Cytometer fluorescence photography. The non-transduced $CD4^+$ T cells (TCRneg-CD4) or PBMC (TCRneg-T) were included as the negative control. The representative fluorescent images and statistical results are shown. One-way ANOVA and Sidak's multiple

comparisons test were used to compare the selected two groups. ***, P < 0.001. The TCR135-CD4 and TCRneg-CD4 groups were shared between Figure 4E and supplementary Figure 5B. **(C)** C666-1-EBNA1 tumor cells were cocultured with TCR135-T cells or TCRneg T cells at the effector-to-target ratio of 1:2 and 1:5, and the growth of tumor cells was assessed by Incucyte real-time quantitative live-cell analysis platforms.

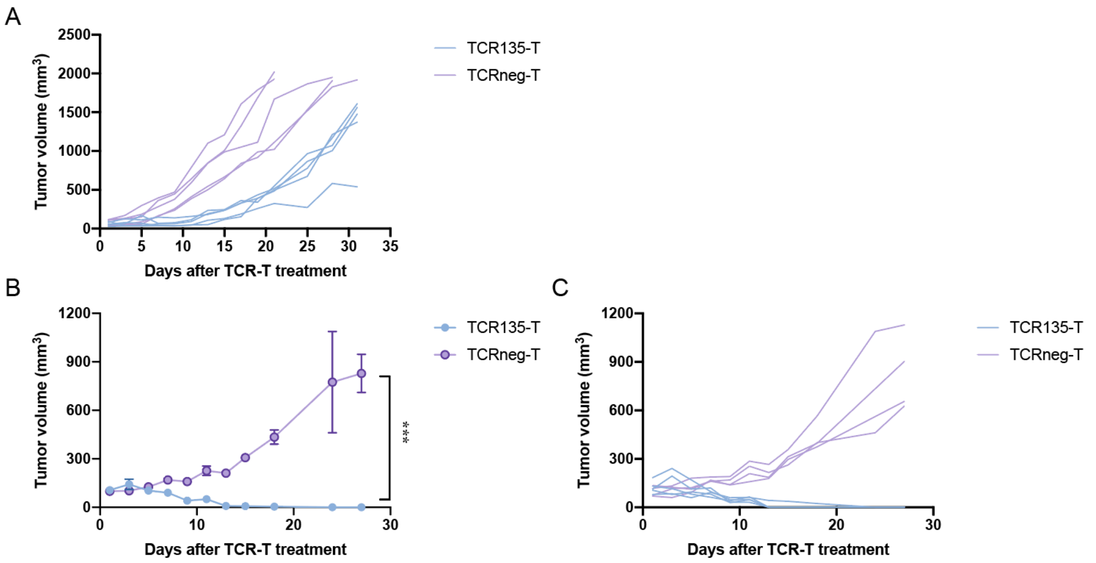

Supplementary Figure 6. (A) The tumor growth curves for each individual mouse in Figure 5C were displayed. **(B-C)** SNU-719-CIITA-EBNA1 tumor cells were subcutaneously injected into NCG mice and treated either with 1×10^7 TCR135-transduced T cells (n = 5) or TCRneg (n = 4) as a control. The mean tumor growth curves for the two groups are shown in (B). Statistical analysis was perfomed using a two-way ANOVA test. ***, P < 0.001. The growth curves for each individual mouse are shown in (C).

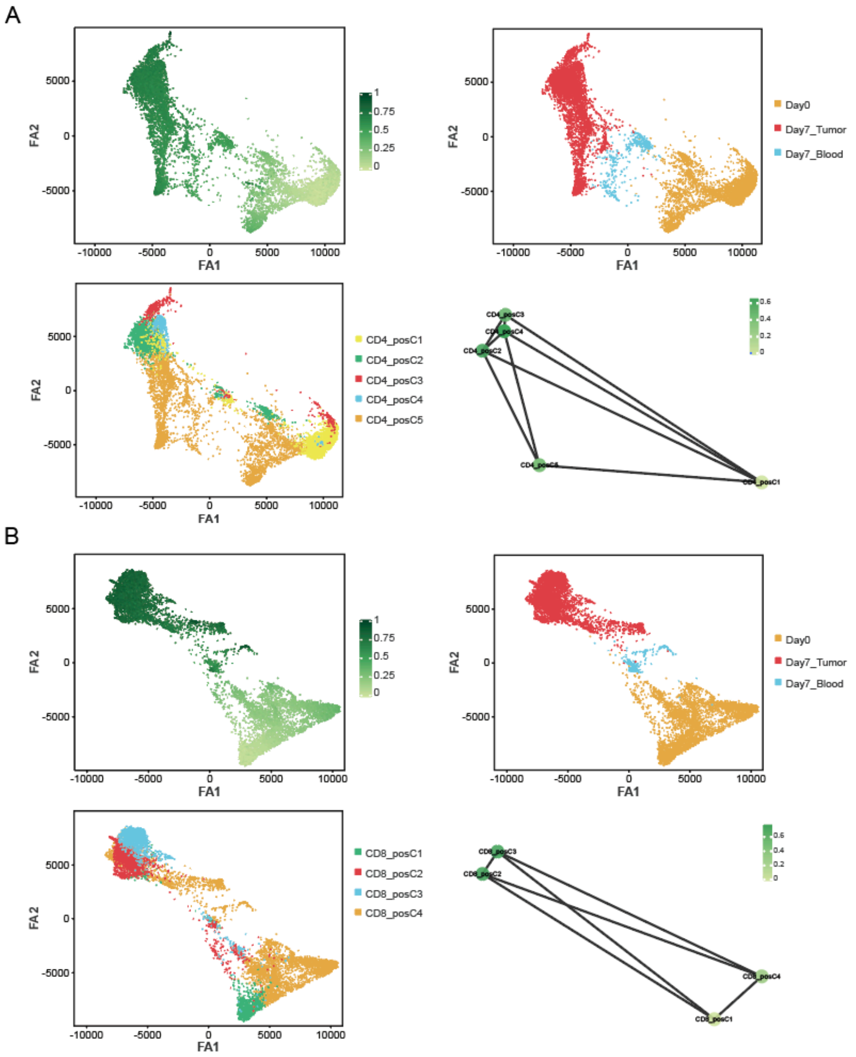

Supplementary Figure 7. Partition-based graph abstraction (PAGA) trajectory analysis of TCR135 positive CD4 (A) and CD8 (B) T cell clusters.

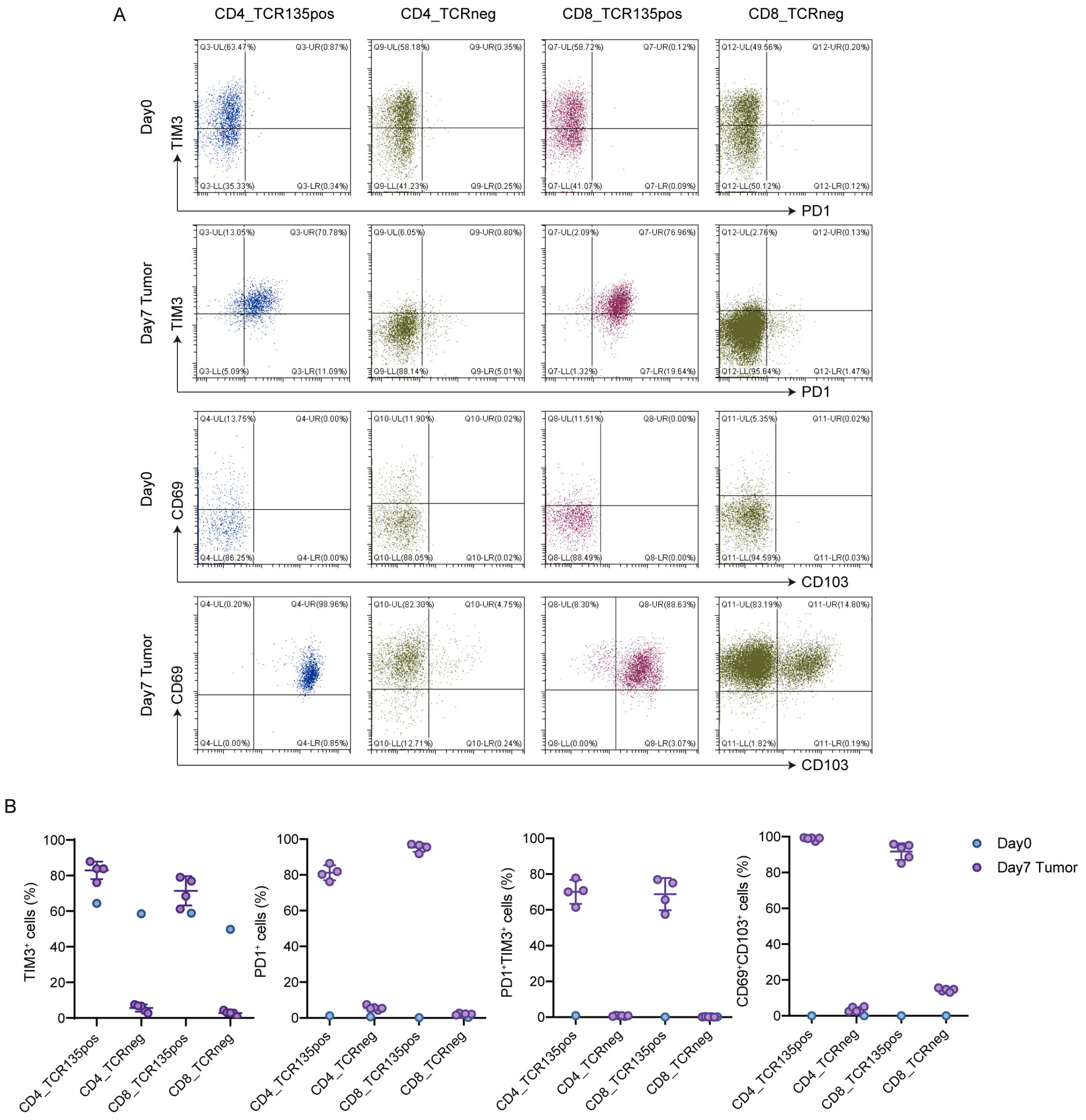

Supplementary Figure 8. TCR135 transduced cells in the tumor microenvironment exhibited upregulated exhaustion markers and tissue-resident memory markers. TCR135-T cells on day 0 before transplantation (Day0) and T cells sorted from tumor tissue (Day7 Tumor) on day 7 after transplantation were analyzed by FACS staining for TIM3, PD1, CD69, and CD103. Representing dot plots are shown in (**A**). The percentage of TIM3, PD1, CD69, and CD103 positive cells in $CD4^+$ and $CD8^+$ T cells are shown in **(B)**.

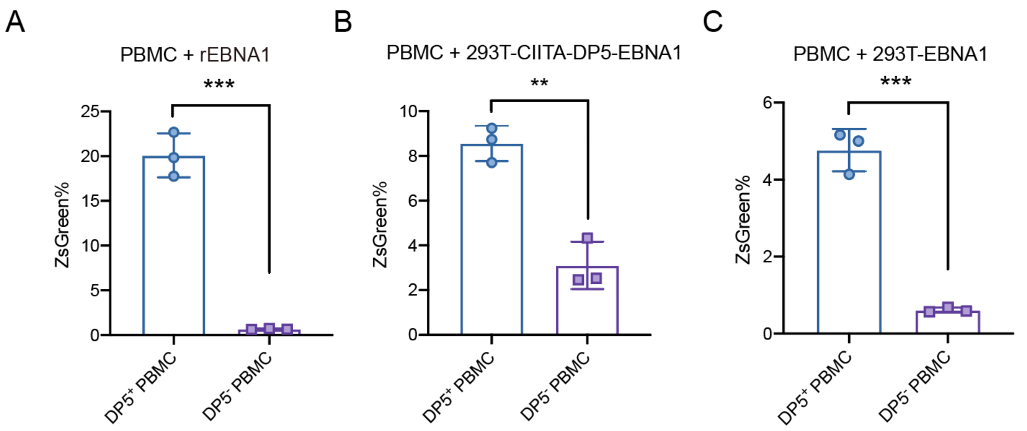

Supplementary Figure 9. TCR135 specifically recognized EBNA1 presented by APCs. TCR135-transduced JK4NF cells were cocultured overnight with DP5^{+}/DP5^{-}-PBMC pulsed with rEBNA1 protein (A), 293T-CIITA-DP5-EBNA1 cell lysate (B), and 293T-EBNA1 cell lysate (C). The frequency of ZsGreen-expressing cells was measured by flow cytometry. Two-tailed unpaired Student's t-test, ***, P < 0.001, **, P < 0.01.

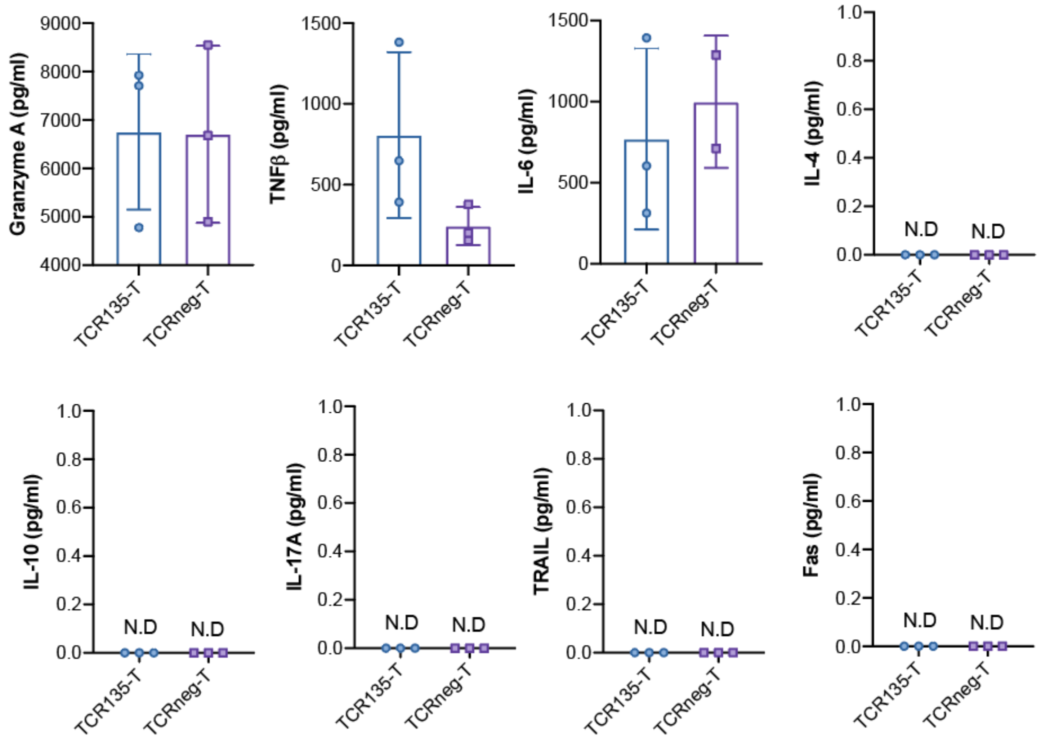

Supplementary Figure 10. The cytotoxic effectors released in the co-culturing supernatant of T cells and rEBNA1-loaded DC were quantified. TCR135-T or TCRneg-T cells were cocultured with rEBNA1-loaded DC for 24 h, then the supernatant was collected, and cytotoxic effectors were detected by RayPlex® Human Cytotoxic T Cell Array Kit 1. N.D, not detected.

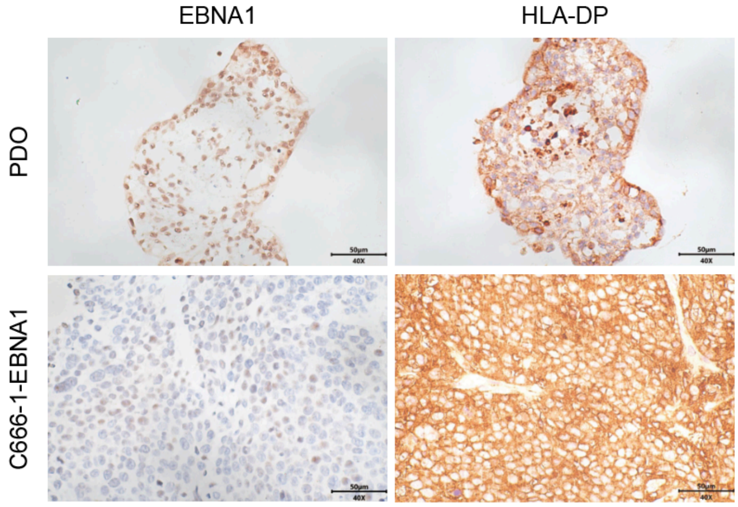

Supplementary Figure 11. The expression of EBNA1 and HLA-DP in PDOs was examined by immunohistochemistry. C666-1-EBNA1 tumor sections were included as the positive control.

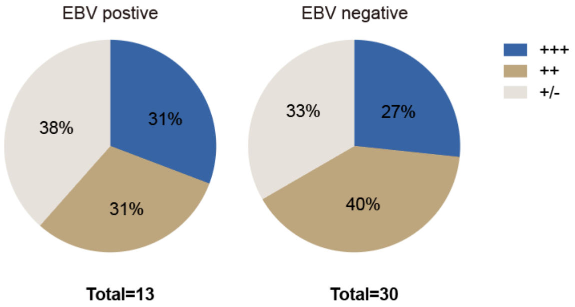

Supplementary Figure 12. The expression of HLA-DP in EBV positive and negative gastric cancers. The paraffin-embedded tissues from 13 EBV-positive and 30 EBV-negative gastric cancer patients were analyzed by immunohistochemistry and were scored by the proportion of HLA-DP$^+$ tumor cells in total tumor cells. The quantitative results are shown in pie charts.

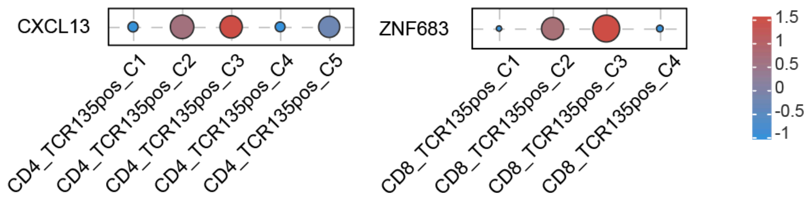

Supplementary Figure 13. The expression of *CXCL13* and *ZNF683* in TCR135pos clusters. The color of the bubbles represents the average gene expression levels, and the size of the bubbles represents the percentage of cells expressing the genes.

Supplementary methods

Production of HLA-DP5 Tetramer. The empty HLA-DP5 monomer was produced as described previously (1). Briefly, pMHC monomers were produced in ExpiCHO cells (Gibco) transduced with HLA-DPB1-p2A-mCherry and HLA-DPA1-p2A-GFP lentiviral vectors. To express DP5-pMHC, the HLA-DP5-ExpiCHO cells were grown in 500 ml flasks (Thermo) in a shaker at 130 rpm, 8% CO_2, and 36.5°C. The basal medium was ExpiCHO Stable Production Medium (Thermo) supplemented with GlutaMAX I (Thermo) and Gibco HT (Thermo). Cultures were started in 80ml of the complete medium at 400,000-500,000 cells/ml and 20% of the original culture volume of the EfficientFeed C+ AGT supplement was added on days 3, 5, 7, 9, and 11. The temperature shifted to 32°C and cells were harvested on day 12.

The DP5 monomer was purified by protein A/G columns and biotinylated in vitro. The biotinylated DP5 monomer was then loaded with peptide by incubation with a 10-fold molar concentration of peptide in 100 mM $NaPO_4$ at pH 6.0, and 0.2% n-octyl-d-glucopyranoside (Macklin) for 72h at 37°C. The peptide-DP5 pMHC molecules were then incubated with APC-streptavidin (Miltenyi Biotec) at a 4:1 molar ratio at 4°C to produce APC-labeled tetramers.

SDS-PAGE. Proteins were electrophoresed in 10% SDS-PAGE gels. To verify the double chain structure of the HLA-DP5 monomer, samples were loaded with and without 20 mM β-ME.

Flow cytometry. Staining of cell surface proteins was done according to standard protocols. For intracellular staining, cells were treated with Cytofix/Cytoperm

Fixation/Permeabilization (BD Biosciences) following standard protocols. Tetramer staining was performed with 10 μg/ml of peptide-pMHC tetramers in PBS or washing buffer for 30 min at 37 ℃. The following fluorochrome-labeled antibodies were purchased from BioLegend: FITC anti-human CD3 (clone UCHT1), FITC/PE anti-human CD4 (clone A161A1), FITC/Pacific Blue anti-human CD8a (clone HIT8a), APC anti-mouse TCRβ chain (clone H57-597), Pacific Blue anti-human IFNγ (clone 4S.B3), PE/Cyanine7 anti-human IL-2 (clone MQ1-17H12), Alexa Fluor 700 anti-human TNFα (clone Mab11), Alexa Fluor 700 anti-human CD154 (clone 24-31), and Alexa Fluor 647 goat anti-mouse IgG. HLA-DP-antibody clone B7/21 was purchased from Abcam. APC-labeled tetramers were produced in our lab. Data were quantified using a BD FACSAria III flow cytometer (BD Biosciences) and CytoFLEX S (Beckman Coulter) and analyzed with FlowJo v. 10 and CytExpert software.

Validation of endogenously presented EBNA1 immunopeptides with LC-MS/MS. The tumor tissues of C666-1-EBNA1$_{\text{C-terminus}}$ cells were harvested and lysed in lysing buffer (20 mM Tris-HCl (pH = 7.5), 150 mM NaCl, 0.5% Triton X-100) for 1 h at 4 °C. Lysates were then spun down with 10,000 g at 4 °C, and supernatant fluids were isolated. For immunopurification of HLA-DP5 ligands, 1 mg of B7/21 antibody was bound to 1 ml of rProtein G Sepharose beads (Cytiva) and incubated with the protein lysate overnight. HLA complexes and binding peptides were eluted using 10% (vol/vol) acetic acid.

The eluates were then loaded on Sep-Pak tC18 cartridges (Waters, 100 mg) and washed with 0.1% TFA and 2% ACN in 0.1% TFA, sequentially. The peptides were

separated from HLA complexes on the cartridges by eluting with 32% ACN in 0.1% TFA and dried using vacuum centrifugation. To confirm the existence of EBNA1 immunopeptides being endogenously processed, both purified native immunopeptides and synthetic peptides were analyzed using an Orbitrap Exploris 480 mass spectrometer coupled to an Ultimate 3000 RSLC nano system (Thermo Fisher Scientific). The samples were reconstituted in 0.1% formic acid and separated on a 25cm column (Aurora) at a flow rate of 300 nL/min using a 90 min gradient of the buffer A (0.1% formic acid) and B (80% ACN and 0.1% formic acid). Data was collected in a parallel reaction monitoring (PRM) mode. The full scan spectra were measured with a resolution of 60,000 within 50 ms maximum injection time, followed by MS2 scans with a resolution of 30,000 within 200 ms maximum injection time. The isolation window of the MS2 scan was set to 1 m/z, and ions within 300 to 100 m/z were triggered for the MS2 event. Acquisition time windows were adjusted individually according to the retention time of their corresponding synthetic peptides. The normalized collision energy was set as 30%. The PRM data was processed with Skyline (version 21.1) software as previously described (2).

Single-cell RNA sequencing and data processing.

Sequencing and quality control

TCR135-transduced T cells from Day 0 were thawed. The $CD45^+$ cells of the tumor and peripheral blood on Day 7 from five TCR135-T treated mice were sorted, pooled, and sent for single-cell RNA sequencing (scRNA-seq). Cellular suspensions were then loaded on a 10X Genomics GemCode single-cell instrument that generates

single-cell Gel Bead-in-Emulsion (GEMs). Libraries were generated from the cDNAs with Chromium Next GEM Single Cell 5' Reagent Kits v3.1 and sequenced by Illumina NovaSeq 6000.

Raw BCL files were converted to FASTQ files, aligned, and count quantified using 10X Genomics Cell Ranger software (version 3.1.0). Cell-by-gene matrices for each sample were individually imported to Seurat (version 3.1.1) for downstream analysis. Cells with an unusually high number of UMIs (⩾8000) or mitochondrial genes (⩾10%) were filtered out. We also excluded cells with less than 500 or more than 4000 genes. Additionally, doublet GEMs were filtered out using the tool DoubletFinder (v. 2.0.3) by using the PC distance to find each cell's proportion of artificial *k*-nearest neighbors (pANN) and ranking them according to the expected number of doublets.

Cell clustering and cell type annotation

The Seurat (3) package was used to perform unsupervised clustering analysis on scRNA-seq data. Briefly, gene counts for cells were normalized by library size and log-transformed. To minimize the effects of batch effect and behavioral conditions on clustering, we used the Harmony algorithm (4) to generate a batch-corrected embedding. An integrated expression matrix was then scaled, and a principal component analysis was used for dimensional reduction. NK cells, NKT cells, $CD4^+$ and $CD8^+$ T cells were defined based on the average expression of signature genes as shown in Supplementary Table 2. $CD4^+$ and $CD8^+$ T cells were processed separately in downstream clustering and signature gene analysis. Uniform manifold approximation and projection (UMAP) was used to visualize clustering results. Bioinformatic analysis was performed using

Omicsmart, a real-time interactive online platform for data analysis (http://www.omicsmart.com).

<u>Calculation of tissue enrichment for cell clusters</u>

The $R_{o/e}$ value was used to estimate the tissue preference of each cell cluster, as previously described (5). $R_{o/e}$ is the ratio of observed cell number over the expected cell number of a given combination of T cell cluster and tissue. The expected cell number for each combination of T cell clusters and tissues was obtained using a chi-squared test (SPSS). $R_{o/e} > 1$ suggests that cells of the given T cell cluster are more frequently observed in the specific tissue than random expectations.

Expression and purification of rEBNA1 protein. The DNA sequence encoding the $EBNA1_{C\text{-terminus}}$ protein with the GST tag was synthesized and cloned into the pGEX vector. *Escherichia coli* BL21(DE3) (Tiangen) transformed with pGEX-GST-EBNA1 was grown in LB medium and protein expression was induced with 1mM IPTG at 16°C overnight. Then the bacteria were harvested and ultrasonically broken. The rEBNA1 protein was purified by glutathione sepharose (GE) columns and identified by MS.

Immunohistochemistry. Tumor samples were fixed in 10% neutral-buffered formalin and embedded in paraffin for sectioning. Tumor tissue section or tissue microarray of NPC (HNasN129Su01, Shanghai Biochip) and gastric cancer (HStmAde150CS401, Shanghai Biochip) were stained with antibodies for human CD3 (Zsbio, clone LN10, ready-to-use), CD4 (Zsbio, clone EP204, ready-to-use), CD8 (Zsbio, clone SP16, ready-to-use), HLA-DPB1 (Abcam, clone EPR11226, 1:2500), and EBNA1 (Abcam, clone E1-2.5, 1:100 or 1:300) according to standard procedures.

Sections were imaged using a Leica Aperio CS2 Slide Scanner and images were processed by Aperio ImageScope software.

Supplementary References

1. Serra P, Garabatos N, Singha S, Fandos C, Garnica J, Sole P, et al. Increased yields and biological potency of knob-into-hole-based soluble MHC class II molecules. *Nat Commun.* 2019;10(1):4917.
2. Chen L, Zhang Y, Yang Y, Yang Y, Li H, Dong X, et al. An Integrated Approach for Discovering Noncanonical MHC-I Peptides Encoded by Small Open Reading Frames. *J Am Soc Mass Spectrom.* 2021;32(9):2346-57.
3. Butler A, Hoffman P, Smibert P, Papalexi E, and Satija R. Integrating single-cell transcriptomic data across different conditions, technologies, and species. *Nat Biotechnol.* 2018;36(5):411-20.
4. Korsunsky I, Millard N, Fan J, Slowikowski K, Zhang F, Wei K, et al. Fast, sensitive and accurate integration of single-cell data with Harmony. *Nat Methods.* 2019;16(12):1289-96.
5. Zhang L, Yu X, Zheng L, Zhang Y, Li Y, Fang Q, et al. Lineage tracking reveals dynamic relationships of T cells in colorectal cancer. *Nature.* 2018;564(7735):268-72.
